# The Atlantic salmon’s stress- and immune-related transcriptional responses to moderate hypoxia, an incremental temperature increase, and these challenges combined

**DOI:** 10.1093/g3journal/jkab102

**Published:** 2021-04-07

**Authors:** Anne Beemelmanns, Fábio S Zanuzzo, Rebeccah M Sandrelli, Matthew L Rise, A Kurt Gamperl

**Affiliations:** Department of Ocean Sciences, Memorial University, St. John’s, NL A1C 5S7, Canada

**Keywords:** warming, hypoxia, environmental changes, acclimation, biomarkers, gene expression, Atlantic salmon, aquaculture

## Abstract

The marine environment is predicted to become warmer, and more hypoxic, and these conditions may negatively impact the health and survival of coastal fish species, including wild and farmed Atlantic salmon (*Salmo salar*). Thus, we examined how: (1) moderate hypoxia (∼70% air saturation) at 12°C for 3 weeks; (2) an incremental temperature increase from 12°C to 20°C (at 1°C week^−1^) followed by 4 weeks at 20°C; and (3) treatment “2” combined with moderate hypoxia affected transcript expression in the liver of post-smolts as compared to control conditions (normoxia, 12°C). Specifically, we assessed the expression of 45 genes related to the heat shock response, oxidative stress, apoptosis, metabolism and immunity using a high-throughput qPCR approach (Fluidigm Biomark™ HD). The expression profiles of 27 “stress”-related genes indicated that: (i) moderate hypoxia affected the expression of several stress genes at 12°C; (ii) their expression was impacted by 16°C under normoxic conditions, and this effect increased until 20°C; (iii) the effects of moderate hypoxia were not additive to those at temperatures above 16°C; and (iv) long-term (4 weeks) exposure to 20°C, with or without hypoxia, resulted in a limited acclimatory response. In contrast, the expression of 15 immune-related genes was not greatly affected until temperatures reached 20°C, and this effect was particularly evident in fish exposed to the added challenge of hypoxia. These results provide valuable information on how these two important environmental factors affect the “stress” physiology and immunology of Atlantic salmon, and we identify genes that may be useful as hypoxia and/or temperature biomarkers in salmonids and other fishes.

## Introduction

Increasing water temperatures, and de-oxygenation of the oceans (hypoxia), as a result of global warming may pose critical challenges to the performance, health and survival of marine organisms (Hoegh-Guldberg and Bruno 2010; McBryan *et al.* 2013; Currie and Schulte 2014; Abdel-Tawwab *et al.* 2019). For example, global ocean temperatures are predicted to increase between 1°C and 3°C by the end of the 21st century (IPCC 2019), and the extent and severity of hypoxic events in shallow coastal waters are intensifying (Breitburg *et al.* 2018; Claret *et al.* 2018; Frölicher *et al.* 2018).

Fish, like other ectotherms, are particularly sensitive to warming because the rate of their biological processes is largely under environmental control (Angilletta *et al.* 2004), and acute and chronic temperature stress can negatively affect their physiological performance (Currie and Schulte 2014; Ern *et al.* 2016; Gallant *et al.* 2017). For commercially farmed Atlantic salmon that are restricted to sea-cages, the most significant environmental challenge is high water temperatures that co-occur with hypoxia during the summer months (Burt *et al.* 2012; Forseth *et al.* 2017; Stehfest *et al.* 2017; Burke *et al.* 2020). In temperate waters of the North Atlantic cultured salmon experience seasonal temperature changes from 0°C to 20°C (this latter temperature observed in late August to early September) (Bjornsson *et al.* 2007; Mansour *et al.* 2008; Burt *et al.* 2012; Burke *et al.* 2020), and Tasmanian salmon in sea-cages experience temperatures as high as ∼23°C during their summer (Stehfest *et al.* 2017; Wade *et al.* 2019). In addition, oxygen levels experienced by salmon inside the sea-cages often decrease to ∼60%–70% air saturation due to several factors including increased oxygen consumption of the fish (*i.e.*, a higher metabolic rate), high-fish density/crowding, low-rates of water exchange, inter-tidal changes and/or algal blooms (Johansson *et al.* 2006, 2007; Beveridge 2008; Oppedal *et al.* 2011; Oldham *et al.* 2018; Solstorm *et al.* 2018; Burke *et al.* 2020). Combined, these suboptimal conditions negatively impact the overall health of farmed Atlantic salmon (McBryan *et al.* 2013; Vikeså *et al.* 2017; Wade *et al.* 2019; Gamperl *et al.* 2020), and they were recently suggested as the primary cause of a mass mortality event in Newfoundland (Burke *et al.* 2020).

Most of our knowledge regarding the effects of temperature and hypoxia on the molecular stress and immune responses of salmonids comes from studies in which fish were exposed to acute temperature changes (over 1–2 hours to days) or acclimated to constant temperatures (Lewis *et al.* 2010, 2016; Quinn *et al.* 2011a; Jeffries *et al.* 2012, 2014; Olsvik *et al.* 2013; Rebl *et al.* 2013; Jørgensen *et al.* 2014; Oku *et al.* 2014; Tomalty *et al.* 2015; Barat *et al.* 2016; Gallant *et al.* 2017; Akbarzadeh *et al.* 2018), or constant low-oxygen conditions (Olsvik *et al.* 2013; Akbarzadeh *et al.* 2020). Furthermore, only one study has looked at the effects of these combined stressors on gene expression in salmonids (Houde *et al.* 2019). Thus, the results of these experiments cannot be extrapolated to the sea-cage environment where the temperature rise is slow and incremental (*i.e.*, at approximately 1°C per week) (Zanuzzo *et al.* 2019) and moderate hypoxia often occurs in combination with increasing temperatures (Burt *et al.* 2012; Burke *et al.* 2020).

Given that multi-stressor experiments are essential to more accurately predicting the effects of foreseen environmental scenarios (Gunderson *et al.* 2016), we recently exposed post-smolt Atlantic salmon to an incremental increase in temperature to 20°C alone, or in combination with moderate hypoxia (∼70% air saturation), and used microarray and qPCR approaches to examine how these two environmental challenges impacted the liver’s transcriptional response (Beemelmanns *et al.* 2021a). Approximately 2900 genes in the liver of these fish were differentially expressed considering both conditions, including those related to the heat shock response, oxidative stress, apoptosis, various metabolic processes and immune function (Beemelmanns *et al.* 2021a). However, this study did not examine at which temperature(s) changes in transcript expression related to the molecular “stress” response and immune function began, and how resilient Atlantic salmon are to long-term high temperature and hypoxia exposure (*i.e.*, do salmon acclimate to these conditions, or do alterations in important processes and pathways intensify?).

In this study, we answered these two questions by measuring transcript expression in the liver of 45 target genes (qPCR, Fluidigm Biomark™ HD) related to the heat shock response, oxidative stress, apoptosis, metabolism and immunity from post-smolt Atlantic salmon of the same experimental groups used in Gamperl *et al.* (2020) and in Beemelmanns *et al.* (2021a, 2021b) when sampled at 12°C, after 3 days at 16°C, 18°C and 20°C, and after 4 weeks at 20°C under conditions of both normoxia and moderate hypoxia. The liver was chosen due to its important role in the stress response, nutrient metabolism and immunity (Richards 2009; Tierney *et al.* 2013), and because it has been effectively used to characterize temperature and hypoxia stress responses in salmon. Our results provide novel insights into the thermal- and hypoxia-sensitivity of “stress” and immune-related transcript expression under simulated cage-site conditions, and we identify genes that might be potential biomarkers for assessing fish health and welfare under these challenging conditions.

## Materials and methods

All experimental procedures described herein were approved by the Institutional Animal Care Committee of Memorial University of Newfoundland (Protocol ^#^16-90-KG) and followed guidelines set by the Canadian Council on Animal Care. Furthermore, all sections of this study adhered to the ARRIVE Guidelines for reporting animal research (Kilkenny *et al.* 2010). This experiment was performed as part of the project “Mitigating the Impacts of Climate-Related Challenges on Salmon Aquaculture (MICCSA),” and a detailed description of the experimental protocol, the fish’s growth characteristics (*e.g.*, specific growth rate, food consumption and feed conversion ratio) and mortality are published in Gamperl *et al.* (2020), while the precise methods used for transcript expression analysis are described in Beemelmanns *et al.* (2021a).

### Animal husbandry and experimental protocol

The experiment was performed from March to August 2017 at the Laboratory for Atlantic Salmon and Climate Change Research (LASCCR), Memorial University, St. John’s, Newfoundland, Canada. Post-smolt Atlantic salmon of Saint John River (NB, Canada) origin obtained from Northern Harvest Sea Farms were randomly distributed into six 2.2 m^3^ indoor fiberglass tanks receiving seawater (32 ppt salinity) at 15 L minute^−1^. The fish were acclimated for four weeks under optimal conditions (∼100%–110% air saturation, 12°C, 32 ppt salinity, 14 hours light: 10 hours dark photoperiod) and were fed a ration of 1% body weight day^−1^ with commercial salmon feed (5 mm, Dynamic S, EWOS Canada Ltd, Surrey, BC, Canada) during this period. All fish in this experiment were implanted with Passive Integrated Transponder (PIT) tags (Loligo Systems; ISO 11784 certified PIT tags, Viborg, Denmark) approximately 2 months before the experiment for identification purposes.

**Figure 1:**
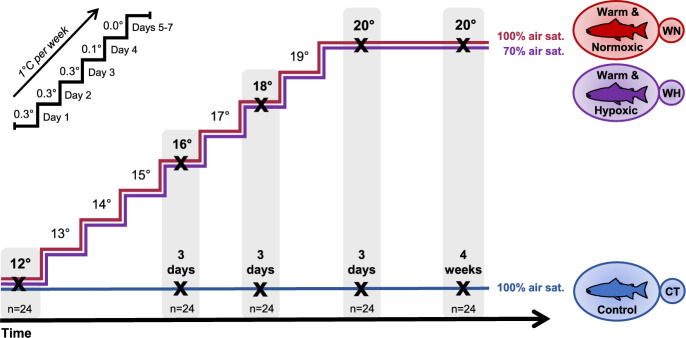
Schematic diagram of the experimental design. Post-smolt Atlantic salmon were either subjected to: (i) a constant water temperature of 12°C and normoxia (100% air saturation) (Control, CT); (ii) a temperature increase from 12°C to 20°C under normoxia (100% air sat.) (Warm & Normoxic, WN); or (iii) moderate hypoxia (∼70% air sat.) and then the incremental increase to 20°C (Warm & Hypoxic, WH). The water temperature was gradually increased by 1°C week^−1^ using the following regimen: 1^st^ day, +0.3°C; 2^nd^ day, +0.3°C; 3^rd^ day, +0.3°C; 4^th^ day, +0.1°C; 5^th^–7^th^ days no increase in temperature (see upper-left portion of the figure). The sampling of liver tissue was performed initially at 12°C, then 3 days after reaching 16°C, 18°C and 20°C, and 4 weeks after the temperature reached 20°C (20°C-4wks) (*n* = 8 per treatment/temperature, *N* = 120 total). The transcriptional responses in the liver of these fish were assessed by measuring the transcript expression of 45 biomarker genes related to the heat shock response, oxidative stress, apoptosis, immunity, metabolism and epigenetics using qPCR (Fluidigm Biomark™ HD).

Three hundred and sixty Atlantic salmon (138.1 ± 1.1 g; mean ± S.E.) were randomly assigned to the following three treatment groups (2 tanks per group; 60 fish tank^−1^) as illustrated in Figure 1. (1) Control (CT): a constant temperature of 12°C and ∼100%–110% air saturation (air sat.) for the duration of the experiment; (2) Warm & Normoxic (WN): incremental temperature increase (12°C–20°C at a rate of 1°C week^−1^) at ∼100% air sat., and then maintained at 20°C for 27 days (∼4 weeks); and (3) Warm & Hypoxic (WH): decrease in water oxygen content to ∼70% air sat. over 1 week, followed by 2 weeks of acclimation to this oxygen level, and then the same temperature regimen (12°C–20°C at a rate of 1°C week^−1^) at 70% air sat. Throughout this experiment, the salmon were carefully fed by hand to satiation twice daily with the commercial salmon feed as indicated above.

In this experiment, fish were sampled at 12°C, 3 days after reaching 16°C, 18°C and 20°C, and finally after 4 weeks at 20°C. For each of the five temperature exposure time points (TP 1–5), we randomly collected four fish per tank, resulting in eight fish per treatment group (*N* = 120 total). Based on *a priori* multiple regression power calculations, and experiments with similar designs, 24 samples at TP 1–5 had sufficient statistical robustness and power (80%) to detect significant differences at *P *<* *0.05 with an estimated medium–large effect size (*f* ^2^ = 0.43) (Beemelmanns *et al.* 2021a). For the sampling procedure, each fish was netted individually from their tank and euthanized by immersion in an aerated seawater bath (∼10 L) containing a lethal dose (0.4 g L^−1^) of the anesthetic MS-222 (tricaine methanesulphonate; Syndel Laboratories, Nanaimo, BC, Canada) followed by cranial concussion. For the current qPCR study, a 200 mg piece of liver was quickly collected from each fish, flash-frozen in liquid nitrogen, and stored at −80°C until further processing.

### RNA extraction and cDNA synthesis

For RNA extraction, 100 mg of flash-frozen liver tissue per sample was homogenized in 800 µl of QIAzol-Lysis Reagent (QIAGEN, Germantown, MD, USA) for 2 minutes at 20 Hz using a TissueLyzerII system with 5 mm stainless steel beads (QIAGEN; Mississauga, ON, Canada) according to the manufacturer’s instructions. To remove potential protein, lipid and genomic DNA contamination, we performed RNA precipitation followed by DNase I treatment and column purification as explained in (Beemelmanns *et al*. 2021a). RNA extraction purity and yields were measured by NanoDrop UV spectrophotometry (NanoDrop; Wilmington, DE, USA), and RNA samples had acceptable A260/280 (2.0–2.2) and A260/230 (1.9–2.3) ratios. Finally, first-strand cDNA templates for qPCR were synthesized in 20 μl reactions from 1 μg of purified total RNA with the QuantiTect^®^Reverse—Transcription Kit (QIAGEN; Mississauga, ON, Canada) following the manufacturer’s protocol.

### qPCR measurements of 45 gene transcripts

#### Gene selection and primer design

We quantified the mRNA expression of 41 genes of interest (GOI) that were pre-selected based on our previous Agilent^®^ 44 K microarray study (Beemelmanns *et al.* 2021a), and of four additional GOIs: 5'adenosine monophosphate-activated protein kinase (*ampk*), apoptosis regulator BAX (*bax*), glucocorticoid receptor 1 (*gr1*) and superoxide dismutase 1 (*sod1*). All of these 45 GOIs were related to one or more of the following functional categories: heat shock response, stress response, oxidative stress response, apoptosis, cellular metabolism, the immune response or transcriptional regulation (DNA methylation) (Table 1). Paralog-specific qPCR primers for these 45 GOIs were designed using the Primer3web platform (v4.1.0; http://bioinfo.ut.ee/primer3/) and were quality tested as described in (Beemelmanns *et al*. 2021a). Only primer pairs with efficiencies between 84% and 108%, and that generated an amplicon product with a single sharp melting curve, were used for qPCR assays (Supplementary Table S1). In addition, three well-established normalizer genes from previous salmon transcriptome studies were included [60S ribosomal protein L32 (*rprl32* and BT043656), eukaryotic translation initiation factor 3 subunit D (*eif3d* and GE777139) and polyadenylate-binding protein 1 (*pabpc1* and EG908498)] (Xue *et al.* 2015; Caballero-Solares *et al.* 2017; Eslamloo *et al.* 2017). Details on the qPCR primer sequences, accession numbers, amplicon sizes and efficiencies can be found in Supplementary Table S1.

**Table 1:** Functional annotation of 45 target genes selected for qPCR analysis

	Gene symbol*^a^*	Gene name^*a*^	Functional category^*b*^	Protein function^*c*^
Temperature- sensitive	*cirbp*	Cold-inducible RNA-binding protein	Cellular stress, hypoxia	RNA stabilization, activator/repressor
	*hsp70*	Heat shock protein HSP 70	Heat shock response	Chaperone
	*hsp90aa1*	Heat shock protein HSP 90-alpha	Heat shock response	Chaperone
	*hsp90ab1*	Heat shock protein HSP 90-beta	Heat shock response	Chaperone
	*hspd1*	60 kDa heat shock protein	Heat shock response	Chaperone
	*serpinh1* (alias *hsp47*)	Serpin H1	Heat shock response	Chaperone

Hypoxia- sensitive	*ampk*	5' adenosine monophosphate- activated protein kinase	Hypoxia, cellular stress, metabolism	Cellular energy homeostasis
	calm (alias *cam*)	Calmodulin	Hypoxia, cellular stress	Calcium ion binding, calcium-signal transduction, response to hypoxia
	*cldn3*	Claudin 3	Hypoxia, cellular stress	Transmembrane signaling, tight junction, response to hypoxia
	*cul3*	Cullin 3	Hypoxia, cellular stress, apoptosis	Ubiquitin–proteasome system, MAPK cascade, HIF1A-pathway
	*egln2* (alias *phd1*)	Egl nine homolog 2	Hypoxia, cellular stress, oxidative stress, apoptosis	Oxygen sensor activity, HIF1A-pathway
	*hif1α*	Hypoxia-inducible factor 1-alpha	Hypoxia, cellular stress, oxidative stress, metabolism	Transcriptional regulator, response to hypoxia
	*igfbp2b1*	Insulin-like growth factor-binding protein 2 precursor	Hypoxia, cellular stress, metabolism	Growth factor binding, signaling pathway

Oxidative stress-related	*cat*	Catalase	Oxidative stress, cellular stress	Oxidoreductase, antioxidant defense
	*cyp1a1*	Cytochrome P450 1A1	Oxidative stress, cellular stress	Oxidoreductase, NADPH electron transport
	*gr1*	Glucocorticoid receptor 1	Cellular stress	Cortisol stress response
	*gstt1*	Glutathione S-transferase theta 1	Oxidative stress, cellular stress	Glutathione metabolic process, antioxidant defense
	*hcn1*	Potassium sodium hyperpolarization-activated cyclic nucleotide-gated channel 1	Cellular stress	Ion transport, mitochondrial respiration
	*jak2*	Tyrosine-protein kinase JAK2	Cellular stress, apoptosis	Protein kinase, apoptosis, singling cascade
	*jund*	Transcription factor Jun-D-like	Cellular stress, immunity, apoptosis	Transcription factor, apoptosis, singling cascade
	*ndufa1*	NADH dehydrogenase 1 alpha subcomplex subunit 1	Oxidative stress, cellular stress	NADH dehydrogenase, mitochondrial respiration
	*ndufa4*	Cytochrome c oxidase subunit NDUFA4	Oxidative stress, cellular stress	NADH dehydrogenase, mitochondrial respiration
	*prdx6*	Peroxiredoxin 6	Oxidative stress, cellular stress	Oxidoreductase, antioxidant defense
	*rraga*	Ras-related GTP-binding protein A	Oxidative stress, cellular stress	GTPase activity, apoptosis, ROS production
	*sod1*	Superoxide dismutase 1	Oxidative stress, cellular stress	Antioxidant defense
	*txn*	Thioredoxin	Oxidative stress, cellular stress	Oxidoreductase
	*ucp2*	Mitochondrial uncoupling protein 2	Oxidative stress, cellular stress	Oxidative phosphorylation, uncoupler activity

Immune- related	*apod*	Apolipoprotein D-like	Immune, inflammation, metabolism	Lipid transporter, response to ROS, inflammation
	*bax*	Apoptosis regulator BAX	Immune response, apoptosis	Apoptosis
	*c1ql2*	Complement C1q-like protein 2	Immune response	Antigen binding, complement classical pathway
	*c3*	Complement C3-like	Immune response, inflammation	Antigen binding, complement alternate pathway
	*camp-a*	Cathelicidin - paralog a	Immune response	Antimicrobial peptide, bacterial defense
	*casp8*	Caspase 8	Immune response, apoptosis	Hydrolase, protease, signaling of apoptosis
	*ctsh*	Cathepsin H precursor	Immune response, apoptosis	Hydrolase, protease
	*epx*	Eosinophil peroxidase-like	Immune response, oxidative stress	Oxidoreductase, peroxidase, inflammatory response
	*il8* (alias *cxcl8*)	Interleukin-8 (Chemokine CXC)	Immune response, inflammation	Cytokine, chemotaxis, inflammatory response, leukocyte migration
	*irf2*	Interferon regulatory factor 2	Immune response	Transcription factor, viral response
	*mhcii* (alias *hla-dra*)	MHC class ii antigen alpha chain	Immune response	Peptide antigen binding, adaptive immunity
	*mmp9*	Matrix metalloproteinase 9	Immune response, apoptosis	Protease, apoptosis, tissue remodeling, wound healing
	*nckap1l*	Nck-associated protein 1-like	Immune response, inflammation, apoptosis	Regulation of phagocytosis, apoptosis
	*tapbp*	Tapasin	Immune response	Peptide antigen binding and presentation
	*tnfrsf6b*	Tumor necrosis factor receptor superfamily member 6 b	Immune response, apoptosis	Signaling receptor, apoptosis

Metabolism	*gck*	Glucokinase	Metabolism	Allosteric enzyme, regulates glucose metabolism
	*pdk3*	Pyruvate dehydrogenase kinase isozyme 3	Metabolism	Protein kinase, regulates cellular glucose homeostasis

Epigenetics	*dnmt1*	DNA (cytosine-5)-methyltransferase 1	Regulation of transcription	Chromatin regulator, DNA methylation

a Refers to the identity of each target gene selected for qPCR (Fluidigm Biomark™ HD). Gene abbreviations are according to UniProt terminology. Further details about primer sequences and primer efficiencies are given in Supplementary Table S1.

b Refers to the broader functional categories for each gene.

c Refers to the protein function according to the UniProt database for each gene.

#### qPCR measurements

The relative transcript expression values of the 45 GOIs and the three reference genes were assessed for the 120 liver samples using the real-time qPCR Fluidigm Biomark™ HD system (Fluidigm; South San Francisco, CA, USA) that is based on a 96.96 Dynamic Array™ IFC (GE-arrays), and according to the protocol described in (Beemelmanns *et al*. 2021a). Briefly, pre-amplification was conducted for each sample by mixing 0.5 µl of a 500 nM primer pool (50 µM primer pair mix) with 2.5 µl of TaqMan-PreAmp Mastermix (Applied Biosystems; Waltham, MA, USA), 0.7 µl of nuclease-free water and 1.3 µl of cDNA (representing 200 ng of input total RNA), and using the following thermal cycler protocol: 10 minutes at 95°C, then 14 cycles of 15 seconds at 95°C and 4 minutes at 60°C. The obtained PCR amplicons were diluted 1:10 with low-EDTA TE buffer (10 mM Tris, 0.1 mM EDTA, pH 8.0). The pre-amplified products were mixed with SSofast-EvaGreen Supermix low Rox (Bio Rad) and DNA-Binding Dye Sample Loading Reagent (Fluidigm) and were loaded along with the primers (50 μM) onto the 96.96 GE-arrays. Then, the GE-arrays were run using the GE Fast 96 × 96 PCR + Melt v2 thermal cycling protocol according to the manufacturer’s instructions (Fluidigm). The transcript expression of the 45 genes was measured in two technical replicates, and we included two no-template controls (NTCs), two controls for genomic DNA contamination (no-reverse transcription “no-RT”), and two linker samples for intra- and inter-run calibration. An efficiency curve (5-point, 3-fold serial dilution) was included to verify the primer efficiencies according to Pfaffl (2001) on this Fluidigm Biomark™ HD system.

#### Data acquisition

Per technical replicate and sample, the mean threshold cycle (*C_T_*), standard deviation (SD), and the coefficient of variation (CV) were calculated. As a quality control for pipetting errors, *C_T_* values (duplicates) with a CV ratio higher than 4% (Bookout and Mangelsdorf 2003) were removed from the data set due to potential measurement error. GeNorm analysis with the qBase+ software (Hellemans *et al.* 2007) was performed on *C_T_* values for the 120 experimental samples. The two normalizer genes *rpl32* (geNorm *M* = 0.302) and *eif3d* (geNorm *M* = 0.313) were identified as the most stable combination (geNorm *V* = 0.115). The RQ of each GOI was determined through normalization to the geometric mean (*C_T_* values) of the two endogenous reference genes (*rpl32* and *eif3d*), including the amplification efficiencies (Supplementary Table S1), and by setting the sample with the lowest expression level as the calibrator sample (*RQ* value = 1.0) (Hellemans *et al.* 2007). The corresponding fold-change (FC) ratios for each GOI were calculated using the raw RQ-values and setting the mean of the CT-group for each exposure temperature point (TP 1–5) as a reference.

### Statistical data analyses

#### Multivariate statistics

All statistical tests and figures were computed in the R environment (v. 3.5.1) (R Core Team 2018). To explore differential expression patterns for the (i) 27 “stress”-related genes (including six temperature-sensitive genes, seven hypoxia-sensitive genes and 14 oxidative stress-related genes) and (ii) 15 immune-related genes (see Table 1), we carried out Principal Component Analysis (PCA) on log_2_ *RQ*-values using the *dudi.pca* function of the *ade4* package in R (Dray *et al.* 2015). For each PCA, the first two principal components (PC-1 and PC-2) were plotted against each other to obtain a projection of the whole data set onto a small dimension, and to account for the most relevant model variance (Nguyen and Holmes 2019). The scores of PC-1 and PC-2 were then extracted, and we fitted a linear mixed-effect model for each of them by applying the *lmer* function of *lme4* (Bates *et al.* 2014) and *lmerTest* packages in R (Kuznetsova *et al.* 2017). Our statistical models were computed with the fixed interaction term “group*temperature”, the covariate “condition factor” and the random term “tank” to account for between-tank variation (*i.e.*, tank effects). For each *lmer* model, the residual distribution and the model fit were examined. Models showing significant results were followed by an estimated marginal means (*emm*) *posthoc* test with the False Discovery Rate (FDR) set at *P *<* *0.05 and using the *emmeans* function in R (Lenth 2016). For each *emmeans posthoc* test, the contrasts were specified to attain pair-wise comparisons between the three treatment groups (CT, WN and WH), within and between each of the five sampling points (12°C, 16°C-3d, 18°C-3d, 20°C-3d and 20°C-4wks). Then, the percentage of the total variance contribution (%) for each gene was evaluated and illustrated in factor maps by applying the *factoextra* package in R (Kassambara 2015). Finally, a hierarchically ordered heatmap was constructed based on log_2_ *RQ*-values of the 45 GOIs using a Pearson correlation and the average UPGMA agglomerative (bottom-up) cluster algorithm.

#### Univariate statistics

The differences in transcript expression between the treatment groups were determined for each gene individually by performing *lmer* models and *emmeans posthoc* tests with the FDR set at *P *<* *0.05 as described in the previous paragraph. Each *lmer* model was graphically examined (histogram and qqplots), and the residuals were tested for normality (Shapiro–Wilk, *P *<* *0.05) and equal variance (Levene test). The *RQ*-values were transformed with either log_2_ or box-cox transformation when necessary to fulfill the assumptions of normality and equal variance.

## Data availability

The obtained threshold cycle (CT) values for the samples of the current study are accessible on-line at the PANGAEA data sharing server (https://doi.org/10.1594/PANGAEA.913696). Supplemental files (Supplementary Tables S1–S4 and Supplementary Figure S1) are available at figshare (https://figshare.com/s/893b1284a406be4aeb38). Supplementary Table S1 contains details about qPCR primers. Supplementary Table S2 contains the complete results of the multivariate statistical approach. Supplementary Table S3 contains the complete results of the univariate statistical approach. Supplementary Table S4 contains the fold-change (FC) ratios of the 45 target genes. Supplementary Figure S1 is a clustered and hierarchical ordered heatmap based on fold-change expression values of the 45 target genes. Supplementary material is available at figshare: https://figshare.com/s/893b1284a406be4aeb38.

## Results and discussion

### High temperature initiates transcriptional “stress” responses in the salmon liver

**Figure 2:**
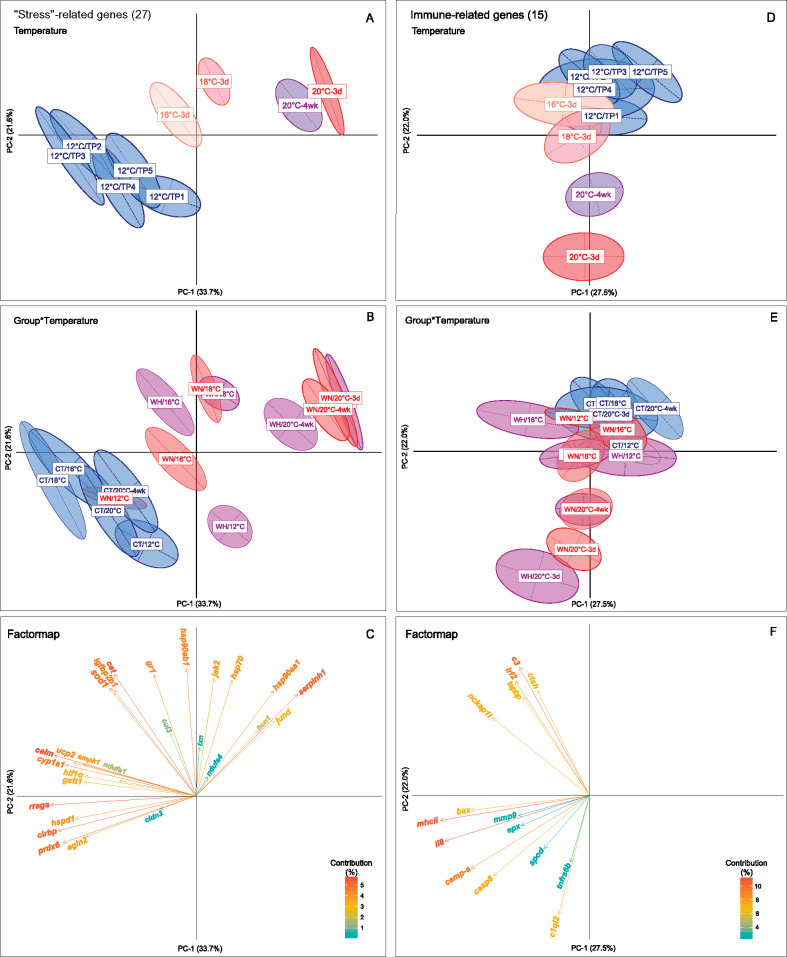
The transcriptional responses of 27 “stress”- and 15 immune-related genes in the Atlantic salmon liver. Principal Component Analysis (PCAs) for (A) 27 “stress”-related genes and (D) 15 immune-related genes based on the transcriptional expression profiles of fish subjected to the five temperature points (TP 1-5) at 12°C, 16°C-3d, 18°C-3d, 20°C-3d and 20°C-4wks alone or combined with moderate hypoxia (∼70% air saturation) (*n* = 8, *N* = 120 total). PCAs for (B) 27 “stress”-related genes and (E) 15 immune-related genes to visualize expression profiles between the three treatment groups (CT, WN and WH) at the five different temperature points (12°C, 16°C-3d, 18°C-3d, 20°C-3d and 20°C-4wks). Each PCA is based on log_2_ *RQ*-values and shows the differential expression between the treatment groups with ellipses that denote the dispersion of variance around the center of the distribution for each group. The variance explained by the main Principal Components (PC-1 and PC-2) is specified as percentage values (%) next to the axes. The statistical results of the temperature and hypoxia treatment effects on the extracted scores of PC-1 and PC-2 are given in Table 2 and Supplementary Table S2. Factor maps representing the contribution of variance (%) explained by (C) 27 “stress”-related genes and (F) 15 immune-related genes that are symbolized by arrows. The length of an arrow pointing to a gene is proportional to the contribution of the variance of this gene to the total variability, and is highlighted with a color gradient. Genes marked in red color tones have a higher contribution (5%–15%), while genes in blue tones have a lower contribution to the variance (0%–5%).

**Figure 3:**
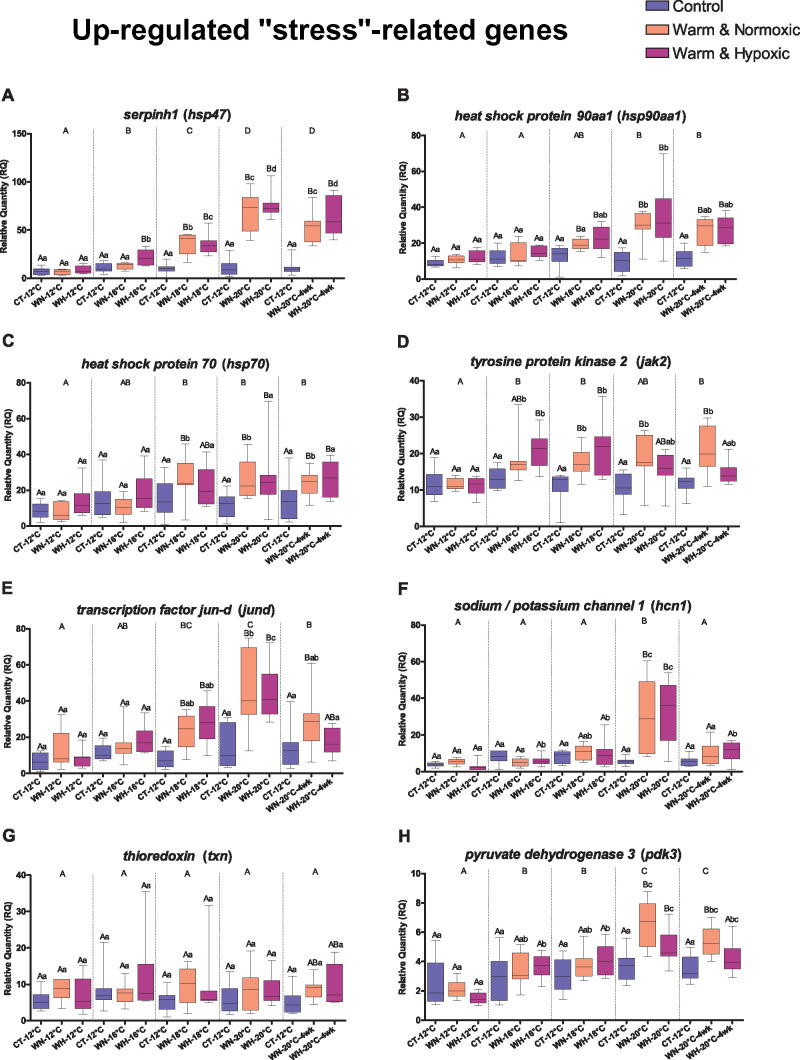
Transcript expression changes for eight up-regulated genes related to the heat shock response, cellular stress, oxidative stress, apoptosis and metabolism. Boxplots show the effects of Control (CT: 12°C, 100% air saturation), Warm & Normoxic (WN: 12°C–20°C, 100% air sat.) and Warm & Hypoxic (WH: 12°C–20°C, ∼70% air sat.) treatments on the RQ of individual genes (*n* = 8, *N* = 120 total). The plots are sorted according to heat shock response (A) *serpinh1*, (B) *hsp90aa1*, (C) *hsp70*; apoptosis (D) *jak2*, (E) *jund*; oxidative stress response (F) *hcn1*, (G) *txn*; and metabolism (H) *pdk3*. The horizontal line within the box indicates the median value, the top and bottom limits of the box indicate the 25^th^ and 75^th^ quartiles, and the bars indicate maximum and minimum values. Dissimilar letters above the error bars of the box plots indicate groups that are significantly different (*emmeans posthoc* test with FDR *P*-value correction, *P *<* *0.05). Capital letters show differences between treatment groups (CT, WN and WH) within a specific temperature (12°C, 16°C-3d, 18°C-3d, 20°C-3d and 20°C-4wks). The lower-case letters represent significant differences comparing a group (CT, WN and WH) across temperatures (12°C, 16°C-3d, 18°C-3d, 20°C-3d and 20°C-4wks). Capital letters on top of the panel indicate differences between the temperature exposures (*emmeans posthoc* test, *P *<* *0.05). The corresponding fold-change values are given in Supplementary Table S4.

Atlantic salmon are cultured in sea-cages and face seasonal water temperature fluctuations (Burt *et al.* 2012; Stehfest *et al.* 2017; Wade *et al.* 2019; Burke *et al.* 2020) that are expected to intensify with climate change (Frölicher *et al.* 2018; Oliver *et al.* 2018; IPCC 2019). Thus, we exposed post-smolt salmon to an incremental temperature increase (12°C–20°C at 1°C week^−1^) that reflects summer conditions in Atlantic Canada (Burt *et al.* 2012; Burke *et al.* 2020). In these fish, increasing water temperatures resulted in the differential expression of 27 “stress”-related genes in the liver (*lmer*-PC-1-Group: *P *=* *0.001; Temperature: *P *<* *0.0001; GP*Temp: *P *<* *0.0001; which explained 33.7% of the variance; Table 2, Figure 2, A–C). Interestingly, the first signs of temperature-induced transcript level changes were detected at 16°C (*emmeans-*WN: 12°C vs. 16°C *P *=* *0.004; Supplementary Table S2, Figure 2B, Supplementary Figure S1), where a significantly different expression profile was found in WN-fish as compared to CT-fish (*emmeans-*16°C: CT vs. WN *P *=* *0.002; Supplementary Table S2, Figure 2B). With a further increase in temperature (18°C–20°C), the WN-fish showed a progressively greater differential expression of these “stress”-related genes with a peak response once temperatures reached 20°C (*emmeans-*WN: 16°C vs. 18°C *P *=* *0.053, 16°C vs. 20°C *P *<* *0.0001, 18°C vs. 20°C *P *<* *0.0001; Supplementary Table S2, Figure 2B, Supplementary Figure S1). The up-regulated genes that primarily contributed to explaining this temperature treatment effect were: *serpinh1, hsp90aa1, hsp90ab1, hsp70, jak2* and *jund* (4%–6% of variance per gene; Figure 2C). Not surprisingly, the highly up-regulated genes *serpinh1, hsp90aa1, hsp90ab1* and *hsp70* (Figure 3 Supplementary Figure S1) encode for heat shock proteins (HSPs), and function as molecular chaperones that maintain cell homeostasis and survival (Basu *et al.* 2002; Roberts *et al.* 2010; Mohanty *et al.* 2018). HSPs guide the folding of newly synthesized proteins, the refolding of misfolded proteins, and the proteolysis of damaged proteins (Parsell and Lindquist 1993; Hartl and Hayer-Hartl 2002). In fish, HSPs are well characterized as protective proteins against thermal stress (Roberts *et al.* 2010) and their mRNA expression varies with acute and chronic temperature changes (Lewis *et al.* 2010, 2016; Evans *et al.* 2011; Quinn *et al.* 2011b; Jeffries *et al.* 2014, 2016; Logan and Buckley 2015; Guo *et al.* 2016; Jesus *et al.* 2016; Li *et al.* 2017; Houde *et al.* 2019; Shi *et al.* 2019). In our study, we found that salmon subjected to WN conditions showed a 3.61-fold higher expression of *serpinh1* (alias *hsp47*, encoding SERPINH1) at 18°C (*P *<* *0.0001) and a 5.51-fold increase at 20°C (*P *<* *0.0001) as compared to CT-fish held at 12°C (Table 3, Supplementary Tables S3, S4 and Figure 3A). Since SERPINH1 plays an important role in collagen biosynthesis and binds collagen molecules to facilitate their assembly and stabilization (Ishida and Nagata 2011), our results suggest an acceleration of processes related to the synthesis and stabilization of collagen molecules in an attempt to maintain hepatocyte structures. Moreover, SERPINH1 is involved in the breakdown of reactive oxygen species (ROS) produced during cellular oxygen stress as recently shown in rainbow trout (*Oncorhynchus mykiss*) (Wang *et al.* 2016b). Hence, the increased expression of *serpinh1* mRNA in the liver may have assisted in the stabilization of collagen molecules within the extracellular matrix (ECM), and further enabled the elimination of generated ROS to maintain cellular homeostasis during this thermal challenge. This hypothesis agrees with what has been observed for Atlantic salmon exposed to 19°C for 21 and 56 days (Jørgensen *et al.* 2014). These fish showed increased *hsp47* expression and collagen I molecules in cardiac tissue, and this response may be indicative of connective tissue remodeling with long-term warm acclimation (Jørgensen *et al.* 2014). Our finding of increased *serpinh1* expression is also consistent with several other studies which report that this gene’s transcript expression is up-regulated after thermal stress in salmonids, and thus, support the use of *serpinh1* as a temperature stress biomarker (Akbarzadeh *et al.* 2018; Houde *et al.* 2019). A similar pattern was observed for *hsp90aa1* (alias *hsp90-alpha* or *hsp90aa*, encoding HSP 90-alpha) which had a 1.51-fold up-regulation at 18°C (*P *=* *0.041) and a 3.08-fold increase at 20°C (*P *<* *0.0001) in WN-fish as compared to CT-fish (Table 3, Supplementary Tables S3 and S4, Figure 3B). This transcript expression suggests that liver HSP90 levels were higher in Atlantic salmon exposed to elevated temperatures, and that this was related to enhanced chaperone protein folding and/or the degradation of misfolded proteins (Jackson 2013). Higher expression of *hsp90aa* in different tissues during hyperthermia is well documented in salmonids (Roberts *et al.* 2010; Jeffries *et al.* 2012, 2014; Rebl *et al.* 2013; Akbarzadeh *et al.* 2018; Houde *et al.* 2019; Swirplies *et al.* 2019). For example, rainbow trout exposed to a more acute increase in temperature from 18°C to 24°C (1°C day^−1^) exhibited ∼27- and 50-fold elevations in *hsp90aa* mRNA in the head kidney and liver, respectively (Li *et al.* 2017; Huang *et al.* 2018). Two Pacific salmon species (*Oncorhynchus nerka* and *O. gorbuscha*) had ∼2-fold increased *hsp90aa1* expression in the gills after 7 days at 19°C as compared to 14°C (Jeffries *et al.* 2014). Houde *et al.* (2019) showed that two paralogues of *hsp90a* (in addition to *serpinh1*) were up-regulated in the Chinook salmon (*O. tshawytscha*) gill when the fish were exposed to 18°C vs. 10°C or 14°C for 6 days. Furthermore, we found that *hsp70* (encoding HSP 70) transcript expression in the WN-group was up-regulated at both 18°C (1.76-fold increase, *P *=* *0.0348) and 20°C (2.04-fold increase, *P *=* *0.0474) as compared to CT-fish at 12°C (Table 3, Supplementary Tables S3 and S4, Figure 3C). HSP70 proteins assist with the folding of nascent polypeptides and the repair or degradation of altered/denatured proteins (Kiang and Tsokos 1998; Basu *et al.* 2002). Thus, increased *hsp70* transcript expression upon exposure to elevated temperatures suggests that there was enhanced chaperone-mediated folding and repair within the salmon’s liver cells, and this agrees with observations for other salmonids (Akbarzadeh *et al.* 2018; Houde *et al.* 2019).

**Table 2:** Temperature and hypoxia treatment effects on the first two Principal Components (PC-1 and PC-2) based on the mRNA expression of 27 “stress”-related and 15 immune-related genes

Treatment effects on the first two principal components (PC-1 and PC-2)^*a*^						
*‘stress’-related genes (27)*	Model	NumDF	DenDF	F.value	Pr(>F)	*Post-hoc* test (*emmeans*)
	*PC-1 (33.7% variance)*					
	Group	8	4	66.61	0.001**	CT vs. WN; CT vs. WH
	Temperature	9	101	25.74	<0.0001***	12°C vs. 20 °C-3d; 12°C vs. 20°C-4wks; 16°C-3d vs. 20°C-3d; 16°C-3d vs. 20°C-4wks; 18°C -3d vs. 20°C-3d; 18°C-3d vs. 20°C-4wks
	Condition factor	11	104	8.20	0.005**	
	Group: temperature	12	101	7.08	<0.0001***	See Supplementary Table S2 for results of all pair-wise comparisons.
	
	*PC-2 (21.6% variance)*					
	Group	8	4	8.52	0.042*	CT vs. WN; CT vs. WH
	Temperature	9	101	2.06	*0.092*	
	Condition factor	11	104	0.48	0.489	
	Group: temperature	12	101	0.91	0.514	

*Immune-related genes (15)*	*PC-1 (27.5% variance)*					
	Group	14	3	1.01	0.451	
	Temperature	15	101	0.81	0.523	
	Condition factor	17	103	0.07	0.796	
	Group: temperature	18	101	0.93	0.495	
	
	*PC-2 (22.0% variance)*					
	Group	14	4	21.15	0.011*	CT vs. WN; CT vs. WH
	Temperature	15	101	15.17	<0.0001***	12°C vs. 20°C-3d; 12°C vs. 20°C-4wks; 16°C-3d vs. 20°C-3d; 16°C-3d vs. 20°C-4wks; 18°C-3d vs. 20°C-3d; 18°C-3d vs. 20°C-4wks; 20°C-3d vs. 20°C-4wks
	Condition factor	17	104	0.87	0.354	
	Group: temperature	18	101	5.14	<0.0001***	See Table S2 for results of all pair-wise comparisons.

a The linear mixed-effect models (*lmer*) were performed on the extracted scores of PC-1 and PC-2 individually to assess the “group*temperature” interaction effect, and included the covariate “condition factor (CF)” and the random term “tank”. The Principal Component Analysis (PCA) was computed based on log_2_ RQ-values for (i) 27 “stress”-related genes and (ii) 15 immune-related genes (Figure 2). The variance explained by each PC is indicated as a percentage (%) value. Significant *lmer* models were followed by *emmeans posthoc* tests with False Discovery Rate (FDR) correction of *P*-values to obtain a pair-wise comparison between Control (CT), Warm & Normoxic (WN) and Warm & Hypoxic (WH) treatments within and between temperatures (12°C, 16°C-3d, 18°C-3d, 20°C-3d and 20°C-4wks). Significant values are marked in bold letters (***P < 0.0001, **P < 0.001, *P < 0.05) and trends in italics (0.05 <*P *<* *0.1). The complete results, containing all values of the obtained pair-wise comparisons, are shown in Supplementary Table S2.

**Table 3:** The expression of 45 target genes for the Warm & Normoxic (WN) and Warm & Hypoxic (WH) treatment groups at each temperature Oxidative stress

Up- or down-regulation of 45 target genes in the WN- and WH-groups at each temperature^*a*^																
		12°C			16°C-3d			18°C-3d			20°C-3d			20°C-4wks		
Gene	Functional category	WN vs. CT	WH vs. CT	WN vs. WH	WN vs. CT	WH vs. CT	WN vs. WH	WN vs. CT	WH vs. CT	WN vs. WH	WN vs. CT	WH vs. CT	WN vs. WH	WN vs. CT	WH vs. CT	WN vs. WH
*serpinh1♦*	Temp. stress					↑	↓↑	↑	↑		↑	↑		↑	↑	
*hsp90aa1♦*	Temp. stress							↑	↑		↑	↑		↑	↑	
*hsp70♦*	Temp. stress							↑			↑	↑		↑	↑	
*hsp90ab1*	Temp. stress															

*cirbp♦*	Temp. stress		↓	↑↓	↓	↓		↓	↓		↓	↓		↓	↓	
*hspd1♦*	Temp. stress		↓	↑↓	↓	↓		↓	↓		↓	↓		↓	↓	

*jak2♦*	Stress, apoptosis				(↑)	↑		↑	↑		↑	↑		↑		↑↓
*jund♦*	Stress, apoptosis							↑	↑		↑	↑		↑		

*hcn1♦*	Oxidative stress										↑	↑				
*txn♦*	Oxidative stress													(↑)	(↑)	
*ndufa4*	Oxidative stress															

*prdx6♦*	Oxidative stress	↓	↑↓	↓	↓		↓	↓		↓	↓		↓	↓	
*ucp2♦*	Oxidative stress				↓	↓		↓	↓		↓	↓		↓	↓	
*rraga♦*	Oxidative stress				↓	↓		↓	↓		↓	↓		↓	↓	
*cyp1a1♦*	Oxidative stress							↓	↓		↓	↓		↓	↓	
*gstt1*	Oxidative stress							(↓)	(↓)		↓	↓		↓	↓	
*ndufa1*	Oxidative stress			↑↓				↓	↓							
*sod1*	Oxidative stress															
*cat*	Oxidative stress															
*gr1*	Oxidative stress															

*egln2*	Hypoxia response							↓			↓	↓		↓	↓	
*calm*♦	Hypoxia response			↑↓	(↓)			↓	↓		↓	↓		↓	↓	
*hif1α*♦	Hypoxia response			↑↓							↓	↓		↓	↓	
*cldn3*	Hypoxia response			↑↓												
*ampk*	Hypoxia response															
*igfbp2b1*	Hypoxia response															
*cul3*	Hypoxia response															

*c1ql2♦*	Immune response							↑	↑		↑	↑			↑	
*casp8♦*	Immune, apoptosis										↑	↑		↑	↑	
*tnfrsf6b♦*	Immune, apoptosis					(↑)		↑	↑			↑		(↑)	(↑)	
*apod♦*	Immune response							↑			↑	↑		↑	↑	
*epx♦*	Immune response							↑			↑	↑		↑	↑	
*camp-a♦*	Immune response											(↑)	(↓↑)	↑	↑	
*il8♦*	Immune response											↑				
*bax*	Immune, apoptosis															
*mhcii*	Immune response															
*mmp9*	Immune response															

*ctsh♦*	Immune response				↓			↓	↓		↓	↓		↓	↓	
*nckap1l*	Immune response										↓	↓				
*c3*	Immune response															
*irf2*	Immune response															
*tapbp*	Immune response															

*gck*	Metabolism				↓	↓		↓	↓							
*pdk3♦*	Metabolism										↑	↑		↑		↑↓

*dnmt1♦*	Epigenetics		↓	↑↓	↓	↓		↓	↓		↓	↓		↓	↓	

a The arrows indicate the direction of the mRNA expression response (up-regulation or down-regulation) and represent the results of the *emmeans post- hoc* test used to obtain pair-wise comparisons between CT, WN and WH treatments across temperatures (12°C, 16°C-3d, 18°C-3d, 20°C-3d and 20°C-4wks). The arrows indicate a significant (*P *<* *0.05) up- or down-regulation when comparing WN vs. CT, WH vs. CT and WN vs. WH, whereas arrows in brackets indicate trends (0.05 < *P *<* *0.1). See Supplementary Table S3 for the results of the linear mixed-effect models for each gene, and Supplementary Table S4 for the exact fold-change ratios.

**Figure 4:**
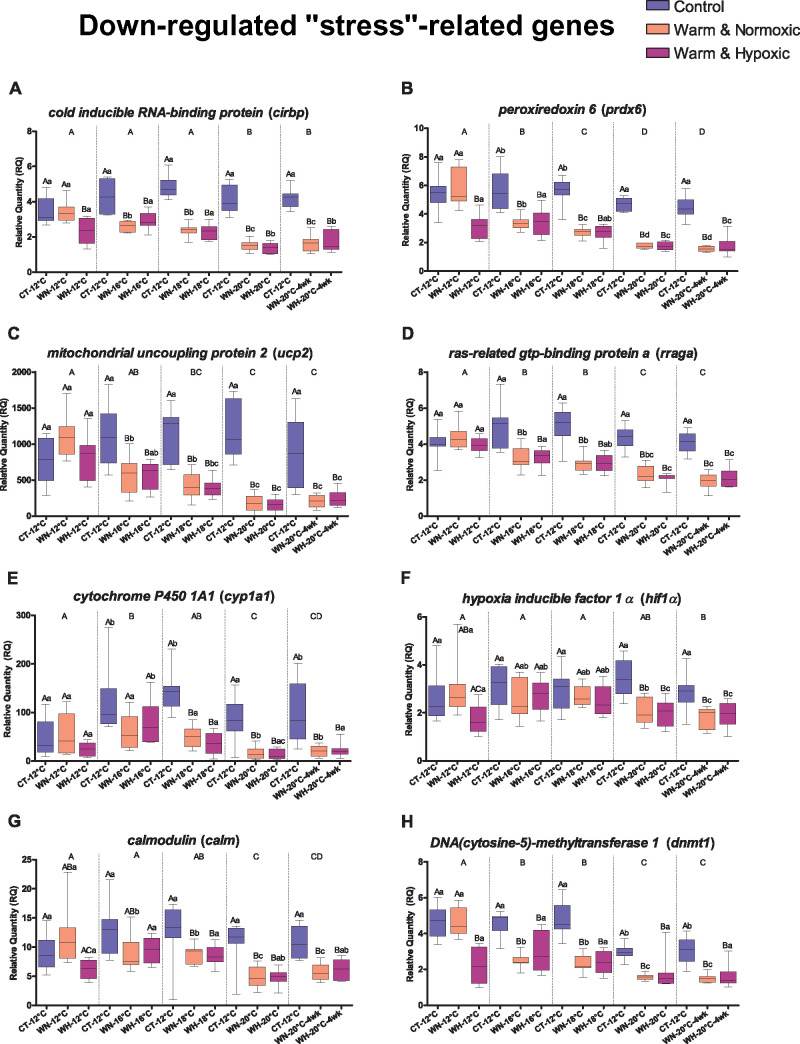
Transcript expression changes for eight down-regulated genes related to hypoxia and oxidative stress responses, and DNA methylation. Boxplots show the effects of Control (CT: 12°C, 100% air saturation), Warm & Normoxic (WN: 12°C–20°C, 100% air sat.) and Warm & Hypoxic (WH: 12°C–20°C, ∼70% air sat.) treatments on the RQ of individual genes (*n* = 8, *N* = 120 total). The plots are sorted according to oxidative stress response (A) *cirbp*, (B) *prdx6*, (C) *ucp2*, (D) *rraga*, (E) *cyp1a1*; hypoxia-response (F) *hif1α*, (G) *calm*; and DNA methylation (epigenetic regulation) (H) *dnmt1.* The horizontal line within the box indicates the median value, the top and bottom limits of the box indicate the 25^th^ and 75^th^ quartiles, and the error bars indicate maximum and minimum values. Dissimilar letters above the error bars of the box plots indicate groups that are significantly different (*emmeans post-hoc* test with FDR *P*-value correction, *P *<* *0.05). Capital letters show differences between treatment groups (CT, WN and WH) within a specific temperature (12°C, 16°C-3d, 18°C-3d, 20°C-3d and 20°C-4wks). The lower-case letters represent significant differences comparing a group (CT, WN and WH) across temperatures (12°C, 16°C, 18°C, 20°C-3d and 20°C-4wks). Capital letters on top of the panel indicate the differences between the temperature exposures (*emmeans post-hoc* test, *P *<* *0.05). The corresponding fold-change values are given in Supplementary Table S4.

**Figure 5:**
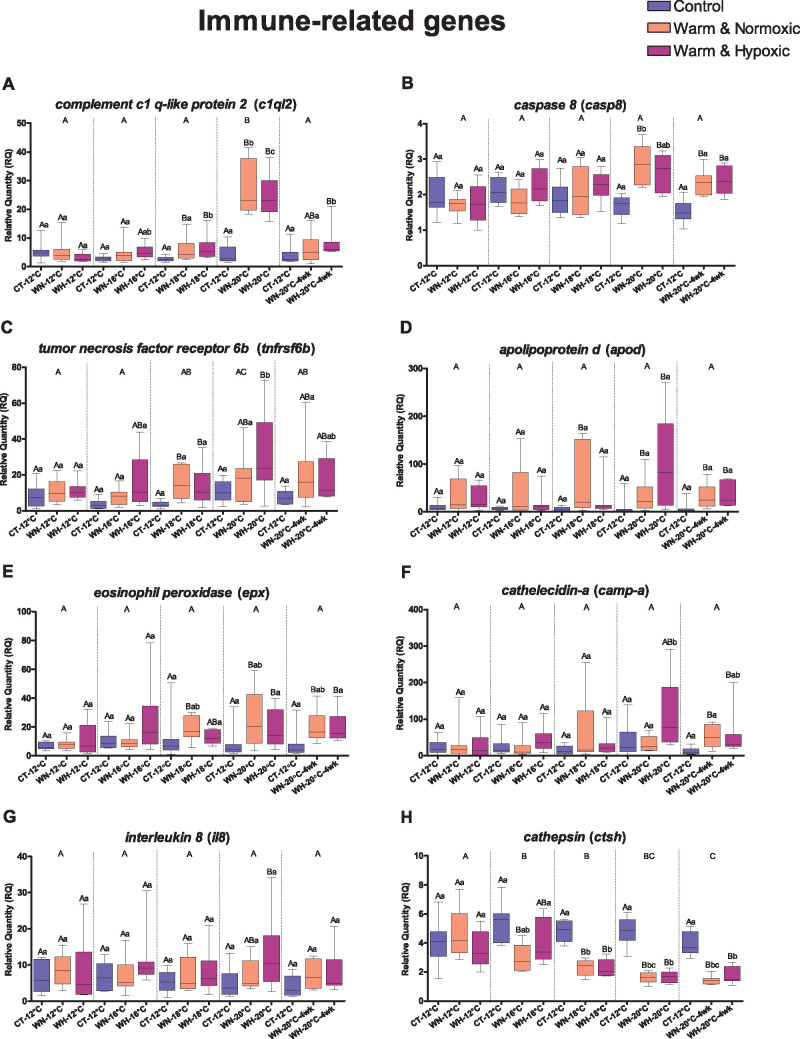
Transcript expression changes for eight immune-related genes. Boxplots show the effects of Control (CT: 12°C, 100% air saturation), Warm & Normoxic (WN: 12°C–20°C, 100% air sat.) and Warm & Hypoxic (WH: 12°C–20°C, ∼70% air sat.) treatments (*n* = 8, *N* = 120 total) on the relative quantity (RQ) of (A) *c1ql2*, (B) *casp8*, (C) *tnfrsf6b*, (D) *apod*, (E) *epx*, (F) *camp-a*, (G) *il8* and (H) *ctsh.* The horizontal line within the box indicates the median value, the top and bottom limits of the box indicate the 25^th^ and 75^th^ quartiles, and the error bars indicate the maximum and minimum values. Dissimilar letters above the error bars of the box plots indicate groups that are significantly different (*emmeans post-hoc* test with FDR *P*-value correction, *P *<* *0.05). Capital letters show differences between treatment groups (CT, WN and WH) within a specific temperature (12°C, 16°C-3d, 18°C-3d, 20°C-3d and 20°C-4wks). The lower-case letters represent significant differences comparing a group (CT, WN and WH) across temperatures (12°C, 16°C-3d, 18°C-3d, 20°C-3d and 20°C-4wks). Capital letters on top of the panel indicate the differences between the temperature exposures (*emmeans post-hoc* test, *P *<* *0.05). The corresponding fold-change values are given in Supplementary Table S4.

♦ Genes that were selected in Figures 3–5 for the illustration of the transcript expression response.

The 1.60-fold up-regulation of the transcript *jak2* (encoding janus kinase 2) at 18°C (*P *=* *0.0057), and 1.73-fold increase at 20°C (*P *=* *0.0048) in WN-fish (Table 3, Supplementary Tables S3 and S4, Figure 3D) indicates that the activity of the JAK/STAT (janus kinase/signal transducers) signaling pathway that promotes cell growth, survival, development and differentiation was stimulated (Rawlings 2004). The JAK/STAT pathway is activated by pro-inflammatory cytokines as well as growth hormone (GH) factors, represents an important signaling pathway for growth processes (Herrington and Carter-Su 2001), and is involved in immune cell signaling (Ghoreschi *et al.* 2009). Thus, cell growth and signaling between immune cells may have been enhanced in WN-fish at 20°C, as further evidenced by an induction of immune-related pathways (see “immune-related gene expression” section below and Beemelmanns *et al.* 2021a).

An activation of anti-apoptotic processes was apparent by the significant 2.89-fold up-regulation of the transcript *jund* (encoding transcription factor JunD) in the liver of WN-fish with warming to 18°C (*P *=* *0.0035), and the 3.18-fold increase at 20°C (*P *=* *0.0001), in comparison to CT-fish (Table 3, Supplementary Tables S3 and S4, Figure 3E). JunD acts as a modulator to protect cells from p53-dependent senescence and apoptosis (Weitzman *et al.* 2000). Indeed, the 1.71-fold up-regulation of *casp8* (encoding caspase 8) in WN-fish at 20°C (*P *=* *0.0001) in comparison to CT-fish at 12°C (Table 3, Supplementary Tables S3 and S4, Figure 5B) indicates that some apoptosis pathways were also induced in the liver cells of these fish (Kruidering and Evan 2000; Redza-Dutordoir and Averill-Bates 2016). The increased levels of HSP transcripts (*i.e.*, *hsp90aa1*) (Figure 3B) could have also been associated with cellular resistance to apoptosis (Lanneau *et al.* 2008). Since exposure to prolonged hypothermia can result in extensive cell death in the liver (*e.g.*, hepatocytes) due to increased oxidative stress (Poon *et al.* 2007; Cheng *et al.* 2015; Liu *et al.* 2016; Li *et al.* 2019), these anti-apoptotic processes may have been induced to counteract the activation of the apoptotic program, and thus, prevent liver necrosis and the loss of liver function (D’Arcy 2019; Wade *et al.* 2019).

An increase in temperature results in higher mitochondrial respiration in fish, including Atlantic salmon (Iftikar and Hickey 2013; Rodnick *et al.* 2014; Chung and Schulte 2015; Gerber *et al.* 2020, 2021). Thus, it was not surprising, that there was a 5.40-fold up-regulation of *hcn1* transcripts in the liver of WN-fish once temperatures reached 20°C (*P *<* *0.0001) in comparison to CT-fish (Table 3, Supplementary Tables S3 and S4, Figure 3F). Hyperpolarization-activated cyclic nucleotide-gated cationic (HCN) channels consist of four family members (HCN 1–4) that contribute to native pacemaker currents and control action potential firing (Ludwig *et al.* 1999). These channels also contribute to the mitochondrial transport of sodium (Na^+^) and potassium (K^+^) ions and the control of the inner mitochondrial membrane potential, and these are connected with the activity of the respiratory chain and ATP synthesis (Santoro and Tibbs 1999; Biel *et al.* 2009; León-Aparicio *et al.* 2019). Mitochondria produce ATP via oxidative phosphorylation and play a vital role in calcium homeostasis and maintenance of cellular redox status (Banh *et al.* 2016). Hence, the large induction of *hcn1* expression at 20°C in the liver cells of WN-fish may have been important in maintaining/elevating mitochondrial energy metabolism under heat stress.

However, accelerated mitochondrial respiration with increasing temperatures also results in a higher mitochondrial ROS production in fishes, which can ultimately cause oxidative stress and cellular dysfunction, apoptosis and tissue damage (Abele *et al.* 2002; Iftikar and Hickey 2013; Rodnick *et al.* 2014; Almroth *et al.* 2015; Chung and Schulte 2015; Gerber *et al.* 2020, 2021). Thus, the activities of enzymes that eliminate harmful ROS and cellular damage and maintain cell homeostasis are often enhanced (Heise 2006; Machado *et al.* 2014). Interestingly, we found marked down-regulation for many genes related to oxidative stress and antioxidant defense in the liver of fish exposed to warming temperatures (12°C–20°C) that also explained a great amount of model variance (5%–6% per gene; Figure 2C); these include *cirbp, cyp1a, gstt1, rraga, prdx6* and *ucp2* (Table 3, Figure 4). For example, the 1.67- and 2.70-fold down-regulation of *cirbp* (encoding cold-inducible RNA-binding protein) transcript expression at 16°C (*P *=* *0.0019) and 20°C (*P *<* *0.0001) in WN-fish (Table 3, Supplementary Tables S3 and S4, Figure 4A) confirms its high sensitivity to hyperthermia, and this finding is consistent with several other temperature studies on salmonids (*e.g.*, Akbarzadeh *et al.* 2018). The expression of *cirbp* has been reported to be induced by cold water, but to be suppressed in response to heat stress; and it encodes a cold-shock protein that acts as an RNA chaperone implicated in multiple cellular processes such as cell proliferation, cell survival, mRNA stability and transcriptional regulation (Zhong and Huang 2017). The 1.67- and 2.63-fold down-regulation of *prdx6* (encoding peroxiredoxin 6) at temperatures from 16°C to 20°C (*P *<* *0.0001) (Table 3, Supplementary Tables S3 and S4, Figure 4B) suggests that these fish had a reduced capacity for lipid peroxidation repair (Fisher 2011; Arevalo and Vázquez-Medina 2018). A down-regulation in *prdx6a* was also observed in the liver of Antarctic emerald rockcod (*Trematomus bernacchii*) exposed to warming temperatures (0°C–25°C) (Tolomeo *et al.* 2016). Furthermore, the temperature increase from 12°C to 20°C resulted in a gradual, but large, decrease in *ucp2* expression in WN-fish (to 5.88-fold at 20°C; *P *<* *0.0001; Table 3, Supplementary Tables S3 and S4, Figure 4C). This gene encodes for mitochondrial uncoupling protein 2 and is a key player in the coupling/uncoupling of oxidative phosphorylation from ATP synthesis (Brand and Esteves 2005). The down-regulation of *ucp2* expression with increasing temperatures agrees with observations in the liver and gill of pikeperch (*Sander lucioperca*) when exposed to an increase from 15°C to 25°C (Swirplies *et al.* 2019). Under stressful conditions, reduced UCP expression would result in increased mitochondrial coupling and a decrease of mitochondrial ROS formation (Laskowski *et al.* 2016). Interestingly, Atlantic salmon acclimated to 20°C have a higher cardiac mitochondrial membrane potential, and reduced cardiac mitochondrial ROS production, when tested at 20°C in comparison to fish acclimated to 12°C (Gerber *et al.* 2020, 2021). The expression of *rraga* mRNA (encoding ras-related GTP-binding protein A) was also decreased by 1.89-fold with warming to 20°C (*P *<* *0.0001) in comparison to CT-fish (Table 3, Supplementary Tables S3 and S4, Figure 4D), and this may be related to the regulation of ROS production and redox/cellular signaling pathways (Finkel 2006; Ferro *et al.* 2012). The Ras GTPase superfamily includes several classes of small GTP-binding and hydrolyzing proteins (GTPases) that act as signal transducers for several pathways responsible for cytoskeletal integrity, cell differentiation, survival and growth, as well as programmed cell death (Ferro *et al.* 2012). In addition, members of the Ras GTPase superfamily can regulate intracellular ROS production and redox signaling, and this strongly contributes to overall cell function (Finkel 2006; Ferro *et al.* 2012). Finally, the 2.56-fold (*P *<* *0.0014) to 5.26-fold (*P *<* *0.0004) down-regulation of the redox gene *cyp1a1* (encoding cytochrome P450 1A1) upon exposure to temperatures between 18°C and 20°C, as compared to 12°C (Table 3, Supplementary Tables S3 and S4, Figure 4E), is consistent with hepatic transcriptional changes reported for Atlantic salmon exposed to 17°C or 19°C for 45 days when compared to 13°C (Olsvik *et al.* 2013). Cytochrome P450s (CYPs) comprise a large gene superfamily of heme-thiolate monooxygenase enzymes that catalyze the oxidation of various organic substances and are considered as biosensors for the environmental monitoring of toxicants (Bistolas *et al.* 2005). Thus, this result suggests that the oxidase system in salmon at elevated temperatures may be compromised, and it could lead to a higher susceptibility to cellular damage if these fish are simultaneously exposed to pollutants (Sokolova and Lannig 2008; Rodgers *et al.* 2021).

Interestingly, the transcript expression levels of the classical antioxidant genes *sod1* (encoding superoxide dismutase 1), *cat* (encoding catalase), and *txn* (encoding thioredoxin) in the WN-group did not differ significantly from those of the CT-group (see Table 3 and Supplementary Table S3, Figure 3G). The proteins SOD and CAT catalyze stepwise reactions involved in the dismutation of superoxide anions to water and hydrogen peroxide, and the reduction of hydrogen peroxide to water, respectively (Halliwell and Gutteridge 2015). These results agree with other fish studies which report that hepatic SOD and CAT mRNA levels are not affected upon prolonged or moderate exposure to elevated temperature (Enzor and Place 2014; Almroth *et al.* 2015), and a recent study from our group showing that neither catalase nor superoxide dismutase activity (in red blood cells and liver) were different when measured in salmon held at 20°C vs. those at 12°C (Zanuzzo et al. 2021). Suppressed gene expression related to oxidation-reduction function after chronic high-temperature stress (19°C vs. 13°C for 45 days) was also observed in the liver of Atlantic salmon by Olsvik *et al.* (2013). Furthermore, a diminished antioxidant defense was evident in the Antarctic bald notothen (*Pagothenia borchgrevinki*) when exposed to an increase in water temperature (+5.6°C) for 3 weeks, and this resulted in the accumulation of oxidative damage in the liver (measured as protein carbonyls and lipid peroxides) (Almroth *et al.* 2015). Clearly, additional research is needed to establish under what conditions, and in which species, chronic temperature increases to near-lethal levels result in increased tissue oxidative damage.

An elevation in circulating cortisol levels is a highly conserved response to stressor exposure in vertebrates, and is essential for animals to overcome and/or adapt to particular stressors (Barton 2002). In our study, the expression level of *gr1* (encoding glucocorticoid receptor 1) was not significantly different in WN-fish as compared to CT-fish (Table 3 and Supplementary Table S3), and this result is consistent with Zanuzzo et al. (2021) who showed that elevated plasma cortisol levels were not encountered until the salmon experienced water temperatures of 21°C or above. Likewise, the salmon in the current study did not show significant differences in their specific growth rate, weight gain or condition factor throughout the experiment (Gamperl *et al.* 2020). These data suggest that the salmon were not stressed by the incremental temperature increase to 20°C, and this agrees with recent data on the growth and physiological stress responses (*i.e.*, body mass, fork length and plasma cortisol levels) of juvenile Atlantic salmon exposed to a long-term increase from 12°C to 20°C (Tromp *et al.* 2018). Collectively, these findings suggest that water temperatures currently experienced in Atlantic Canada during the summer/early fall (*i.e.*, 18°C–20°C) do not cause significant phenotypic changes (*i.e.*, growth performance) or induce a physiological stress response (*i.e.*, based on plasma cortisol levels) in salmon of Saint John River stock. However, the increased expression of several genes (*e.g.*, *serpinh1*, *hsp90aa* and *hsp70*) or decreased (*e.g.*, *ucp2* and *cirbp*) at temperatures beginning at ∼16°C indicates that these fish mounted a robust cellular stress response to cope with these conditions.

### High temperature initiates immune-related transcript expression changes in the salmon liver

The incremental temperature increase under normoxic conditions (WN) resulted in the differential expression of 15 immune-relevant genes measured in the salmon liver (*lmer*-PC-2-Group: *P *=* *0.011; Temperature: *P *<* *0.0001; Group*Temperature: *P *<* *0.0001; explaining 22% of the variance; Table 2, Figure 2, D–F). However, this differential expression was highly temperature dependent. There was only a slight impact on immune transcript expression at temperatures of 18°C or below (*emmeans-*18°C: CT vs. WN *P *=* *0.044; Supplementary Table S2, Figure 2E), while a strong response was induced once temperatures reached 20°C (*emmeans-*20°C: CT vs. WN *P *<* *0.0001; *emmeans-*WN: 16°C vs. 20°C *P *<* *0.0001, 18°C vs. 20°C *P *=* *0.001; Supplementary Table S2, Figure 2E). Exposure to high temperatures has previously been shown to affect immune-related transcript expression and constitutive immunity in several fish species (Lewis *et al.* 2010; Rebl *et al.* 2013; Xu *et al.* 2013; Jeffries *et al.* 2014; Oku *et al.* 2014; Tomalty *et al.* 2015; Barat *et al.* 2016; Jesus *et al.* 2016). In this study, one of the key responsive immune genes was *c1ql2* (c1q-like protein 2), which had a 6.39-fold higher expression in the liver of WN-fish once temperatures reached 20°C (*P *<* *0.0001) as compared to CT-fish (Table 3, Supplementary Tables S3 and S4, Figure 5A). This suggests that the classical complement system was activated, possibly for the detection and elimination of bacterial pathogens (Holland and Lambris 2002), but also indicates that the adaptive immune response was activated as C1q binds to the Fc portion of aggregated antibody-antigen (IgG and IgM) immune complexes on bacterial surfaces (Nayak *et al.* 2012). Similar results have been reported for rainbow trout at 22°C as compared to 14°C (Oku *et al.* 2014), and acclimation to 20°C for 57 days increased the lytic activity of the total complement system in the plasma of this species (Nikoskelainen *et al.* 2006). Hypothermic temperatures in humans have also been shown to increase antibody-initiated complement activation and eukaryotic cell destruction, which may even contribute to the mechanism of ischemia-reperfusion injury (Shah *et al.* 2014).

The previously mentioned 1.71-fold upregulation of *casp8* expression in WN-fish at 20°C (*P *=* *0.0001) in comparison to CT-fish at 12°C (Table 3, Supplementary Tables S3 and S4, Figure 5B) suggests activation of the death receptor (extrinsic) pathway of apoptosis by ROS, and increased cell death at high temperatures (Kruidering and Evan 2000; Redza-Dutordoir and Averill-Bates 2016). Caspase-mediated apoptosis also involves death receptors from the tumor necrosis factor receptor (TNF-R) superfamily (Redza-Dutordoir and Averill-Bates 2016). Indeed, the 4.51-fold up-regulation of *tnfrsf6b* (encoding TNF-R superfamily member 6B) at 18°C (*P *=* *0.026), but not at 20°C (*P *=* *0.228), in WN-fish as compared CT-fish at 12°C (Table 3, Supplementary Tables S3 and S4, Figure 5C), suggests that protective mechanisms were initiated against apoptosis through the suppression of the FasL- and LIGHT-ligand mediated apoptosis pathways (Yu *et al.* 1999).

The 5.30-fold higher expression of *apod* (encoding apolipoprotein d) with warming to 20°C (*P *=* *0.0228) as compared to 12°C (Table 3, Supplementary Tables S3 and S4, Figure 5D) may be connected to induced chemotactic and proteolytic antimicrobial, and tempering inflammation processes (Crespo-Sanjuán *et al.* 2017). In addition, ApoD and its orthologs have been shown to prevent lipid peroxidation accumulation and an overexpression can protect against neurodegeneration during stress conditions including hyperoxia and heat stress (Muffat *et al.* 2008). In concordance, the 3.09-fold higher expression of *epx* transcripts (eosinophil peroxidase) at 20°C (*P *=* *00042) in WN-fish relative to CT-fish at 12°C (Table 3, Supplementary Tables S3 and S4, Figure 5E) not only suggests the activation of leukocytes (specifically the eosinophils), but also the release of lysosomal enzymes with peroxidase activity and the production of nitric oxide (NO) and/or chlorines/chloramines with bactericidal activity (Jong *et al.* 1980). These results are consistent with our previous findings that fish from the same experiment showed an enhanced leukocyte respiratory burst activity in the blood at 20°C as compared to CT-fish at 12°C (Zanuzzo *et al.* 2020). Phagocytosis and peroxidase release by leukocytes in response to pathogens is a powerful immune mechanism in fish to clear infections (Nikoskelainen *et al.* 2006).

In our previous study, we found that fish of the WN group (20°C for 4 weeks) generally had up-regulated constitutive levels of *il1β*, *il8-a, cox2, hamp-a, stlr5-a* and *irf7-b* at 20°C, and when these fish were challenged with a multivalent vaccine (*Forte V II; containing bacterial* and *viral components/antigens*), they induced similar, and even hastened, innate immune responses as compared to CT-fish at 12°C (Zanuzzo *et al.* 2020). Collectively, these findings indicate that salmon exposed to an incremental temperature increase have a higher constitutive immunity at 18°C–20°C and that high temperature did not weaken, but rather activated/enhanced, their immune system (immunocompetence). This response might have been triggered due to the potential for more virulent pathogens and higher pathogen abundance in warmer water (Vezzulli *et al.* 2010; Kimes *et al.* 2012), or may be associated with alterations in gut bacterial abundance or diversity that can occur at high temperatures (Hagi *et al.* 2004; Neuman *et al.* 2016). The latter could have also influenced their innate immune responses (Gómez and Balcázar 2008; Zanuzzo *et al.* 2020). Still, this research area deserves further attention, and live pathogen exposure experiments need to be conducted to assess the immunocompetence of Atlantic salmon and their susceptibility to infections under predicted IPCC climate change scenarios.

### Moderate hypoxia (∼70% air sat.) at 12°C impacts hypoxia and “stress”-related transcript expression in the salmon liver, but not that of immune-related genes

In this experiment, we subjected salmon to moderate hypoxia ∼70% air sat. (daily range of ∼65%–75% air sat.) as this oxygen level is similar to summer conditions in coastal sea-cages in Newfoundland (Burt *et al.* 2012; Burke *et al.* 2020), and that salmon choose this level of water oxygenation in sea-cages when exposed to the combined challenges of elevated temperatures and low-oxygen levels (Stehfest *et al.* 2017). Interestingly, the WH-fish used in this study had a diminished growth performance (weight gain and specific growth rates) when acclimated to moderate hypoxia at 12°C that was not associated with lower feed consumption (Gamperl *et al.* 2020). In addition, a follow-up study showed that Atlantic salmon of a similar age exposed to moderate hypoxia (∼70% air sat.; 12°C) for 2 weeks had ∼3-fold higher plasma cortisol levels (Zanuzzo et al. 2021), which indicates a physiological stress response (Barton 2002). In the current study, salmon initially acclimated to moderate hypoxia (∼70% air sat.) at 12°C for approximately 3 weeks before the thermal challenge had a significantly different “stress”-related transcript expression as compared to individuals that were maintained at normoxic (∼100% air sat.) conditions at 12°C (*emmeans-*12°C: CT vs. WH *P *=* *0.032, WN vs. WH *P *=* *0.007; Supplementary Table S2, Figure 2B). In contrast, the same low-oxygen treatment did not trigger changes in immune transcript expression (*emmeans-*12°C: CT vs. WH *P *=* *0.819, WN vs. WH *P *=* *0.581, Supplementary Table S2, Figure 2E), and genes with hypoxia response (*hif1α, calm, cld3*) and cellular stress response (*cirbp, prdx6, hspd1* and *ndufa1*) functions were significantly down-regulated in WH-fish as compared to WN-fish (Table 3, Supplementary Tables S3 and S4, Figure 4). Amongst them, the expression of *hif1α* (alias *hif-1a*, encoding hypoxia-inducible factor 1-alpha) was 1.45-fold lower in WH-fish in comparison to CT-fish, and differed significantly from the 1.19-fold up-regulation in WN-fish at 12°C (*P *=* *0.0287) (Table 3, Supplementary Tables S3 and S4, Figure 4F). HIF1-A is a master hypoxia-responsive transcriptional regulator with oxygen sensing functions that regulates cellular processes such as energy metabolism, apoptosis and cell proliferation (Wenger 2002; Kilic *et al.* 2007; Richards 2009). In several fish species, the expression of *hif1α* is generally up-regulated in response to acute and severe hypoxia (Rahman and Thomas 2012; Rimoldi *et al.* 2012; Froehlich *et al.* 2015), while chronic hypoxia exposure results in tissue-specific and exposure time-dependent alterations of *hif1α* mRNA levels (Rimoldi *et al.* 2012; Olsvik *et al.* 2013; Rahman and Thomas 2017). For instance, Rimoldi *et al.* (2012) showed that *hif1α* mRNA was down-regulated in the muscle of Eurasian perch (*Perca fluviatilis*) when exposed to chronic hypoxia (15 days at 30% DO), whereas its expression was not affected in the liver. Similarly, Olsvik *et al.* (2013) did not detect a significant change in *hif1α* expression in the liver of Atlantic salmon when exposed to 4–5 mg O_2_ L^−1^ for 120 days at 12°C. In this study, the significant down-regulation of *hif1α* at 12°C might have been related to the limited period of hypoxia exposure (3 weeks) and/or the moderate nature of the hypoxia. A quite similar expression profile was observed for *calm* (alias *cam*, encoding calmodulin, or calcium-modulated protein), which was 1.45-fold down-regulated in WH-fish as compared to CT-fish at 12°C, and differed significantly from the 1.29-fold up-regulation in WN-fish (*P *=* *0.0115) (Table 3, Supplementary Tables S3 and S4, Figure 4G). Calmodulin is a calcium sensor that controls a large number of enzymes and ion channels in eukaryotic cells by binding calcium (Ca^2+^), and it regulates many crucial processes such as growth and cell proliferation (Chin and Means 2000). The activity of Ca^2+^/calmodulin-dependent protein kinase (CaMK) is affected by hypoxia (Yuan *et al.* 2005), and this pathway is down-regulated in the liver of the hypoxia tolerant gynogenetic blunt snout bream (*Megalobrama amblycephala*) (Gong *et al.* 2020). Hence, the Ca^2+^/CaMK cell signaling pathway may have played a crucial role in mediating hypoxia acclimation in our salmon. Furthermore, hypoxia at 12°C resulted in a 1.41-fold down-regulation of the gene *cirbp* in WH-fish as compared to CT-fish at 12°C (*P *=* *0.0198) (Table 3, Supplementary Tables S3 and S4, Figure 4A). This result is comparable to the previously reported decrease in *cirbp* transcript expression in the hippocampus of rats after exposure to chronic hypoxia (21 days) (Chen *et al.* 2017a). Under stressful conditions, CIRBP interacts with HIF-1α, and is involved in chronic hypoxia-induced apoptosis by indirectly regulating *hif1α* expression through the direct modulation of microRNAs (Chen *et al.* 2017a). Thus, the change in its expression may reflect its evolutionarily conserved role during hypoxia acclimation. Finally, the exposure to moderate hypoxia at 12°C induced a 1.75-fold down-regulation of *prdx6* (*P *<* *0.0001) (Figure 4B). Peroxiredoxin 6 is important in phospholipid homeostasis, lipid peroxidation repair and inflammatory signaling (Fisher 2011; Arevalo and Vázquez-Medina 2018), and may have been an important factor in the oxidative stress response during hypoxic acclimation.

Collectively, these findings demonstrate that chronic exposure to moderate hypoxia (∼70% air sat.) at 12°C was a mild stressor for post-smolt Atlantic salmon, and suggest that it likely initiated molecular and physiological stress-related pathways in the salmon’s liver cells.

### Increasing temperatures (12°C–20°C) in combination with moderate hypoxia (∼70% air sat.) did not impose additive effects on the molecular “stress” response in the salmon liver

Salmon confined in sea-cages endure increasing water temperatures accompanied by decreased oxygen levels in the summer (Oppedal *et al.* 2011; Burt *et al.* 2012; Stehfest *et al.* 2017; Wade *et al.* 2019). To simulate these environmental conditions more realistically, we exposed salmon to warming temperatures (12°C–20°C) combined with moderate hypoxia (∼70% air sat.). These hypoxic fish showed a gradual shift in the expression of “stress”-related transcripts with increasing temperatures in comparison to CT-fish maintained constantly at 12°C (*emmeans-*16°C: CT vs. WH *P *=* *0.028, 18°C: CT vs. WH *P *<* *0.0001; 20°C: CT vs. WH *P *<* *0.0001; *emmeans-*WH: 16°C vs. 18°C *P *=* *0.029, 16°C vs. 20°C *P *<* *0.0001, 18°C vs. 20°C *P *<* *0.0001; Supplementary Table S2, Figure 2B). However, the overall “stress”-related transcript expression of WH-fish was not synergistically, nor additively, affected when compared to the expression profiles of WN-fish, and the fish of both warmed groups shared very similar expression profiles (*emmeans-*16°C: WN vs. WH *P *=* *0.303; 18°C: WN vs. WH *P *=* *0.861; 20°C: WN vs. WH *P *=* *0.672; Supplementary Table S2, Figure 2B). For example, the hypoxic fish had a similar magnitude of differential expression for the previously determined hypoxia-sensitive genes (*cirbp, calm, hif1α, hspd1, ndufa1* and *prdx6*) as temperature increased from 12°C to 20°C as compared to normoxic fish (Table 3, Supplementary Tables S3 and S4, Figure 4). Nonetheless, the WH-fish did experience an up-regulation of two specific stress indicator genes (*serpinh1* and *jak2*) after the initial temperature increases to 16°C (Table 3, Supplementary Tables S3 and S4, Figure 3, A and D). For instance, *serpinh1* was 2.00-fold higher expressed in hypoxic fish after warming to 16°C (*P *=* *0.0267) as compared to the marginal 1.21-fold increase in normoxic fish at 16°C (*P *=* *0.4798) (Figure 3A), and this suggests an earlier activation of chaperone-mediated collagen processing and biosynthesis (Ishida and Nagata 2011). In addition, the 1.56-fold higher expression of *jak2* only in WH-fish at 16°C as compared to CT-fish at 12°C (*P *=* *0.0217) (Figure 3D), suggests a greater promotion of cell survival and proliferation through GH signaling (Rawlings 2004; Reindl *et al.* 2011). With further increases in temperature, the expression profiles of the WN- and WH-groups became very similar (Figure 2, B and E and Supplementary Figure S1). These findings confirm our previous transcriptome study in which the same WN- and WH-fish showed similar transcriptional patterns for ∼2900 genes (Beemelmanns *et al.* 2021a).

Most biological processes in fish are impacted by the abiotic factors of temperature and oxygen as they strongly influence metabolic rate and growth performance (Hevrøy *et al.* 2013; Kullgren *et al.* 2013; Olsvik *et al.* 2013; Vikeså *et al.* 2017; Leeuwis *et al.* 2019; Wade *et al.* 2019). In the current experiment, the WH-fish were smaller, and had a reduced specific growth rate and feed conversion efficiency, in comparison with WN- and CT-fish (Gamperl *et al.* 2020). The suppression of genes related to metabolism has been reported in several fish species after hypoxia (Gracey *et al.* 2001; Zhong *et al.* 2009; Li *et al.* 2018) or high temperature exposure (Olsvik *et al.* 2013; Jeffries *et al.* 2014; Jesus *et al.* 2016; Prado-Lima and Val 2016; Chen *et al.* 2017b; Li *et al.* 2017, 2019). In this study, we report that the expression of *pdk3* [encoding pyruvate dehydrogenase kinase 3, an essential regulator for glycolysis and the maintenance of glucose homeostasis (reviewed in Kuntz and Harris 2018)] was up-regulated by 1.34-fold in WH-fish as compared to CT-fish at 20°C (*P *=* *0.0104) (Table 3, Supplementary Tables S3 and S4, Figure 3H); which was not different from that of the high-temperature treatment group alone. However, *gck* (encoding glucokinase) expression was decreased by 5.26-fold in WN-fish (*P *=* *0.0126) and by 7.14-fold in WH-fish (*P *=* *0.0246) at 18°C as compared to 12°C (Table 3, Supplementary Tables S3 and S4). This latter result suggests that the phosphorylation of glucose to glucose-6-phosphate is reduced in the liver of fish (Wilson 1994) at high temperatures under normoxic or hypoxic conditions. In response to high temperatures, the re-allocation of energy resources is an essential strategy, and when Atlantic salmon are exposed to higher temperatures (18°C–20°C) for prolonged periods the mobilization of liver lipid storage is initiated (Hevrøy *et al.* 2013), and salmon show a decline in plasma amino acids (glutamine, tyrosine and phenylalanine) (Kullgren *et al.* 2013). Indeed, the 5.3- and 9.4-fold up-regulation of *apod* transcripts in WN- and WH-fish at 20°C respectively, suggest that there was increased ApoD mediated transportation of lipids and other small hydrophobic molecules for lipid metabolism (Crespo-Sanjuán *et al.* 2017) in heat-stressed salmon. Our, previous analysis (using the Agilent^®^ 44 K microarray) revealed that the same WN- and WH-fish (at 20°C for 3 days) showed a marked down-regulation of pathways connected to the metabolism of carbohydrates, proteins, fatty acids and lipids in the liver, and that the expression of 19 biomarker genes strongly correlated with seven health parameters in this group (Beemelmanns *et al.* 2021a). Collectively, these findings imply that these transcriptional responses may be associated with a metabolic suppression that resulted in an impairment of physiological and growth performance.

### Increasing temperatures (12°C–20°C) and moderate hypoxia (∼70% air sat.) have additive effects on salmon liver immune-related genes

Salmon acclimated to moderate hypoxia and exposed to an incremental temperature increase to 18°C only experienced minor effects on immune-related transcript expression (*emmeans-*18°C: CT vs. WH *P *=* *0.044; Supplementary Table S2, Figure 2E), whereas the increase to 20°C resulted in a more extreme response and a distinct expression profile in comparison to fish maintained at 12°C (*emmeans-*20°C: CT vs. WH *P *<* *0.0001; Supplementary Table S2, Figure 2E). The overall immune transcript expression of WH-fish was generally similar to that of WN-fish at temperatures above 18°C (*emmeans-*18°C: WN vs. WH *P *=* *0.848; 20°C: WN vs. WH *P* = 0.182; Supplementary Table S2, Figure 2E). However, three immune genes (*i.e.*, *tnfrsf6b,* *camp-a and il8*) were more strongly affected in WH than WN-fish as compared to CT-fish. For example, the expression of *tnfrsf6b* was 1.29-fold higher in WH-fish as compared to WN-fish at 20°C (*P *=* *0.0103) (Figure 5C), and this suggests a stronger inhibition of Fas-ligand-induced apoptosis (Yu *et al.* 1999). Furthermore, the 3.17-fold higher expression of *camp-a* in WH-fish as compared to WN-fish at 20°C (*P *=* *0.0534) (Figure 5F) suggests that there was a considerably greater induction of the antimicrobial peptide (AMP) cathelicidin. The expression of AMPs in fish is initiated due to the presence of microbial stimuli (bacterial DNA and proteins) and they are known to have broad-spectrum antimicrobial activity (Masso-Silva and Diamond 2014; Katzenback 2015). In salmonids, bacterial infections result in an enhanced expression of cathelicidins (Chang *et al.* 2006) with bactericidal and immunomodulatory activity (Bridle *et al.* 2011). The expression of *il8* was also 2.63-fold higher in WH-fish at 20°C (*P *=* *0.0421) as compared to CT-fish, whereas there was no difference between WN- and CT-fish (Table 3, Supplementary Tables S3 and S4, Figure 5G), and this points to the activation of inflammatory responses during warm and hypoxic conditions since the chemotactic factor IL-8 recruits and activates neutrophils, basophils and T-cells to the site of infection (Mukaida 2000). Exposure to high temperature or hypoxia also promotes an increase in the number of leukocytes and in respiratory burst in fish plasma (Nikoskelainen *et al.* 2006). Thus, it is likely that exposure to high temperatures increased the number of circulating leukocytes, and the additional challenge of hypoxia stimulated a higher leukocyte respiratory burst and innate immune response (Zanuzzo *et al.* 2020). As warmer temperatures usually promote microbial growth, they can lead to *Vibrio* infections in marine organisms (Vezzulli *et al.* 2010) because of temperature-dependent mechanisms that can trigger *Vibrio* pathogenicity (Kimes *et al.* 2012). For example, salmon subjected to increasing temperatures (up to 21°C) have an increased abundance of potentially more virulent *Vibrio* spp. in their digestive system, while beneficial lactic acid bacteria (LAB) and *Acinetobacter* spp. disappear (Neuman *et al.* 2016). Hence, our results suggest that the combination of a temperature increase to 20°C and moderate hypoxia resulted in a greater impact on the salmon’s hepatic immune transcript expression and immune competence that may have been beneficial against virulent pathogens in warmer water.

### What is the capacity of Atlantic salmon to tolerate high temperatures of 20°C with or without hypoxia for an extended period?

In this study, we subjected Atlantic salmon to a possible future North Atlantic summer environmental scenario by maintaining 20°C under either normoxia or moderate hypoxia for a total of 27 days (∼4 weeks), a period that would naturally correspond to August and early September on the east coast of Canada. Interestingly, we observed a slightly lower impact on transcript expression changes in the warmed groups after long-term exposure to 20°C, and that overall, the transcript expression of 27 “stress”-related (Figure 2, A–C) and 15 immune-related genes (Figure 2, D–F) returned closer to initial levels. These data suggest that the salmon has some (but limited) capacity to physiologically acclimate to chronic high temperature. For a few genes, we found a pattern of reduced transcriptional response after 4 weeks at 20°C (*i.e.*, *hcn1, c1ql2* and *jund*). More specifically, the gene *hcn1* returned toward initial levels after prolonged exposure to 20°C with moderate hypoxia (Figure 3F). Given that mitochondrial density in the heart of Atlantic salmon was not affected by acclimation to 20°C vs. 12°C (Gerber *et al.* 2021), the down-regulation of *hcn1* suggests that respiration was reduced to maintain mitochondrial energy metabolism in the hepatocytes and could be a key metabolic acclimation response (Santoro and Tibbs 1999; Biel *et al.* 2009; Strobel *et al.* 2013; León-Aparicio *et al.* 2019). A similar pattern was observed for the immune gene *c1ql2* which had a 4-fold lower expression in the liver of WN-fish and a 10-fold lower expression in WH-fish after 4 weeks at 20°C as compared to 3 days at this temperature (Supplementary Table S4; Figure 5A). Hence, it appears that the activity of the classical complement component pathway was reduced in the liver of WN- and WH-fish after long-term stress exposure. Interestingly, the hemolytic activity of the complement system (alternative pathway) and plasma lysozyme concentration were unaffected in these fish (Zanuzzo *et al.* 2020).

Overall, immune transcript expression in the liver of hypoxic fish challenged for 4 weeks at 20°C was less impacted as compared to the initial 3 day exposure at 20°C, although it was still slightly different from hypoxic fish at 18°C (*emmeans-*WH: 20°C-3d vs. 20°C-4wks *P *=* *0.003; 18°C-3d vs. 20°C-4wks *P *=* *0.049; Supplementary Table S2, Figure 2E). We did not observe significant mortalities in the WN or WH treatments after being maintained at 20°C for 4 weeks (Gamperl *et al.* 2020), and when these fish were immune challenged with a multivalent vaccine (*Forte V II; containing both bacterial and viral components/antigens*) their capacity to mount an innate immune response was not impaired in comparison to fish maintained at 12°C (Zanuzzo *et al.* 2020). This suggests that long-term acclimation to high temperatures does not compromise the innate immune responses of these fish (Zanuzzo *et al.* 2020). Interestingly, Jørgensen *et al.* (2014) reported a higher abundance of transcripts for genes involved in innate cellular immunity, but a lower abundance of transcripts related to humoral immunity, in the cardiac tissue of adult salmon exposed to 19°C for 8 weeks. This provides evidence that long-term exposure to high temperatures can influence components of the immune system in some tissues, which could potentially lead to a higher immunocompetence in Atlantic salmon.

There was a trend suggesting that prolonged exposure to WH exposure reduced “stress”-related transcript expression (*emmeans-*WH: 20°C-3d vs. 20°C-4wks, *P *=* *0.092; Supplementary Table S2, Figure 2B). For example, the mRNA expression of *jund* (*P *=* *0.0025) was significantly reduced in hypoxic fish maintained for 4 weeks at 20°C, while it remained up-regulated in WN-fish (Table 3, Supplementary Tables S3 and S4, Figure 3E). As previously stated, JunD is an important transcriptional regulator of a signaling cascade that protects cells from apoptosis and promotes cell survival (Weitzman *et al.* 2000). Since the gene *casp8*, a key regulator of caspase-mediated apoptosis (Kruidering and Evan 2000), remained 1.56-fold up-regulated in WH-fish as compared to CT-fish after 4 weeks at 20°C (*P *=* *0.0027) (Table 3, Supplementary Tables S3 and S4, Figure 5B), a simultaneous lower expression of *jund* may have resulted in reduced protection against apoptosis. These latter results suggest that WH-fish may have suffered more from hepatocyte apoptosis (Poon *et al.* 2007; Cheng *et al.* 2015; Liu *et al.* 2016; Li *et al.* 2019) or a negative impact on the liver function (Wade *et al.* 2019), and this deserves further investigation.

Nevertheless, the expression of 13 target genes related to the heat shock response (*serpinh1, hsp90aa1, hsp70* and *hspd1*), oxidative stress response (*cirbp, cyp1a1*, *gstt1, prdx6, rraga* and *ucp2*) and hypoxic signaling (*calm, egln2* and *hif1α*) were comparable in WN- and WH-fish exposed to 20°C for 3 days vs. 4 weeks (Table 3, Supplementary Tables S3 and S4, Figures 3, 4 and Supplementary Figure S1). For example, the genes *serpinh1* (WH: 5.50-fold, WN: 4.77-fold; *P *<* *0.0001, Figure 3A) and *hsp90aa1* (WH: 2.31-fold, WN: 2.27-fold; *P *<* *0.0001, Figure 3B) remained significantly up-regulated in both groups after 4 weeks at 20°C as compared to CT-fish at 12°C. This suggests that the cellular heat shock response was still activated, and was needed for the maintenance of correct protein folding and to prevent damaged proteins from accumulating (Basu *et al.* 2002; Roberts *et al.* 2010; Mohanty *et al.* 2018). Similarly, Jørgensen *et al.* (2014) showed that the expression of cardiac HSP genes (*i.e.*, *serpinh1, hsp90aa1* and *hsp70*) and collagen I, was still up-regulated in Atlantic salmon exposed to 19°C for 21 and 56 days.

In summary, long-term (4 weeks of) exposure to 20°C did not result in increased transcriptional responses. Rather the investigated immune and “stress”-related response pathways (*i.e.*, apoptosis, mitochondrial respiration and complement component pathway) either remained differentially expressed, or partially returned to basal (12°C) levels. Overall, it appears that fish that experienced the combined stressors of high temperature and low oxygen levels had transcript expression levels that were similar to those of 18°C fish. It is unclear whether this was because they had partially acclimated to the conditions of high temperature (20°C) and hypoxia, or whether prolonged exposure to these conditions led to the exhaustion of particular cellular responses/pathways.

### Epigenetic regulation of transcript expression—DNA methylation

Plastic phenotypic responses facilitating acclimatization to changing environments can be mediated by DNA methylation through the modulation of transcript expression (Duncan *et al.* 2014; Eirin-Lopez and Putnam 2019). Here, we report that *dnmt1* (encoding DNA methyltransferase 1) expression was 2.13-fold reduced in salmon challenged with moderate hypoxia (∼70% air sat.) at 12°C as compared to fish under normoxia (100% air sat.) (*P *<* *0.0001), but was not further impacted by warming to 20°C (Table 3, Supplementary Tables S3 and S4, Figure 5H). Whereas salmon subjected to increasing water temperatures from 12°C to 20°C under normoxia showed a gradual decrease in *dnmt1* transcript expression as compared to CT-fish at 12°C (*P *<* *0.0001) that reached a similar magnitude of down-regulation by 20°C (2.04-fold) (Table 3, Supplementary Tables S3 and S4, Figure 5H). DNMT1 is an enzyme that catalyzes the reversible addition of a methyl group (CH_3_) to the 5' carbon end of cytosine (5mC) and is a key player in the epigenetic regulation of transcript expression (Edwards *et al.* 2017). Interestingly, DNA methylation changes can be rapidly induced by environmental factors (Angers *et al.* 2010), and recent research provides convincing evidence that teleost DNA methylation is influenced in various ways by temperature (Campos *et al.* 2013; Anastasiadi *et al.* 2017; Burgerhout *et al.* 2017; Ryu *et al.* 2020) and hypoxia (Wang *et al.* 2016a; Veron *et al.* 2018). In a complementary study, we found that high-temperature exposure to 20°C alone, or in combination with hypoxia, induced varied changes in the DNA methylation of specific cytosines (CpGs) located within important genomic regulatory elements (*i.e.*, promoter, 5’UTR, 1st exon and 1st intron) of five of the herein investigated treatment responsive genes (*serpinh1, jund, cirbp, prdx6* and *ucp2*), and that these alterations correlated with transcript expression changes (Beemelmanns *et al.* 2021b). These DNA methylation patterns were also highly dynamic and dependent on the duration of exposure. However, persistent changes in specific CpG sites after exposure to 20°C for 4 weeks strongly indicate that these are important “epimarkers” that facilitate thermal acclimation responses (Beemelmanns *et al.* 2021b). Thus, the down-regulation of *dnmt1* in the liver of salmon, when exposed to moderate hypoxia alone and/or with temperature increase from 12°C to 20°C, suggests that genome-wide changes in DNA methylation status likely played a role in mediating the herein observed transcriptional acclimation responses.

## Conclusions and perspectives

In summary, we identified extensive transcriptional changes in 27 “stress”-related and 15 immune-related genes in the liver of post-smolt Atlantic salmon of Saint John River stock when exposed to an incremental temperature increase (12°C–20°C; at 1°C week^−1^) alone, or in combination with moderate hypoxia (∼70% of air sat.); conditions that simulate summer conditions in salmon aquaculture sea-cages in Canada and the North Atlantic (Burt *et al.* 2012; Burke *et al.* 2020). We found that a slow and moderate temperature increase from 12°C to 16°C was sufficient to increase the expression of “stress”-related genes, and that these transcriptional responses intensified as the temperature was increased to 20°C. The suite of target genes included those related to the heat shock response (*serpinh1, hsp90aa1, hsp70* and *hspd1*), apoptosis (*jund, jak2, tnfrsf6b, casp8* and *ctsh*), oxidative and/or general stress responses (*cirbp, prdx6, ucp2, rraga, gstt1, cyp1a1, ndufa1* and *hcn1*) and that are responsive to hypoxia (*calm*, *egln2* and *hif1α*). In contrast, the overall expression of 15 immune-related genes was only impacted when temperatures reached 20°C, and the responsive genes were mainly associated with innate immunity and apoptosis (*c1ql2, casp8, epx, apod, il8, ctsh* and *nckap1*). Interestingly, moderate hypoxia alone impacted transcript expression in the liver of salmon (*calm, dnmt1, hspd1, hif1α* and *prdx6*) at 12°C, and this suggests that this condition triggered minor cellular stress or acclimation responses. However, the transcriptional responses of these genes were not additive or synergistic when hypoxia-acclimated salmon were subsequently challenged with an incremental temperature increase to 20°C. In fact, these transcript expression responses were of a similar magnitude. On the other hand, the overall expression of 15 immune-related genes was more strongly impacted in the liver of hypoxia-acclimated fish subjected to the temperature increase to 20°C (*e.g.*, *camp-a, il8* and *tnfrsf6b*); thus reflecting a higher state of constitutive immunity. Finally, after long-term (4 weeks) exposure to 20°C, the fish showed a trend toward reduced stress and immune transcript expression. Collectively, our data indicate that salmon can adjust their physiology to increasing temperatures, but have some, yet limited, capacity to acclimate to temperatures as high as 20°C. This may not be surprising as mortality of these salmon started to occur when temperatures reached 22°C with ∼30% of mortality by 23°C (Gamperl *et al.* 2020); and the acclimation temperature of 20°C is within a few degrees of the maximum rearing/stocking temperature for this species (Hvas and Oppedal 2019).

Taken together, our results provide valuable information on how these two important environmental challenges affect the stress physiology and immunity of Atlantic salmon, and identify several genes that can be used as biomarkers to characterize the transcriptional stress response with much greater sensitivity as compared to standard physiological stress measures (*e.g.*, plasma cortisol) (Zanuzzo et al. 2021). In future studies, a combination of functional genomics, epigenetics, proteomics, lipidomics, metabolomics, and physiological stress measurements should be utilized to gain a more detailed characterization of thermal tolerance in Atlantic salmon. Toward this end, the MICCSA research group is presently developing ELISAs (Enzyme-Linked Immunosorbant Assays) for several biomarkers identified in this research, including SERPINH1, CIRBP, PRDX6 and IL-8. Such research will be extremely valuable for understanding, and potentially mitigating, the potential impact of these two co-occurring environmental stressors on Atlantic salmon in aquaculture, and for the conservation and management of wild fish populations.
